# An expanded transcriptome atlas for *Bacteroides thetaiotaomicron* reveals a small RNA that modulates tetracycline sensitivity

**DOI:** 10.1038/s41564-024-01642-9

**Published:** 2024-03-25

**Authors:** Daniel Ryan, Elise Bornet, Gianluca Prezza, Shuba Varshini Alampalli, Taís Franco de Carvalho, Hannah Felchle, Titus Ebbecke, Regan J. Hayward, Adam M. Deutschbauer, Lars Barquist, Alexander J. Westermann

**Affiliations:** 1 Helmholtz Institute for RNA-based Infection Research, Helmholtz Centre for Infection Research, Würzburg, Germany; 2 Environmental Genomics and Systems Biology Division, Lawrence Berkeley National Laboratory, Berkeley, CA USA; 3 Department of Plant and Microbial Biology, University of California, Berkeley, Berkeley, CA USA; 4 Faculty of Medicine, University of Würzburg, Würzburg, Germany; 5 Department of Biology, University of Toronto Mississauga, Mississauga, Ontario Canada; 6 Institute of Molecular Infection Biology, University of Würzburg, Würzburg, Germany; 7 Department of Microbiology, Biocentre, University of Würzburg, Würzburg, Germany; 8 Present Address: Department of Radiation Oncology, Technical University of Munich, School of Medicine, Klinikum rechts der Isar, Munich, Germany

**Keywords:** Microbiology, Biochemistry

## Abstract

Plasticity in gene expression allows bacteria to adapt to diverse environments. This is particularly relevant in the dynamic niche of the human intestinal tract; however, transcriptional networks remain largely unknown for gut-resident bacteria. Here we apply differential RNA sequencing (RNA-seq) and conventional RNA-seq to the model gut bacterium *Bacteroides thetaiotaomicron* to map transcriptional units and profile their expression levels across 15 in vivo-relevant growth conditions. We infer stress- and carbon source-specific transcriptional regulons and expand the annotation of small RNAs (sRNAs). Integrating this expression atlas with published transposon mutant fitness data, we predict conditionally important sRNAs. These include MasB, which downregulates tetracycline tolerance. Using MS2 affinity purification and RNA-seq, we identify a putative MasB target and assess its role in the context of the MasB-associated phenotype. These data—publicly available through the Theta-Base web browser (http://micromix.helmholtz-hiri.de/bacteroides/)—constitute a valuable resource for the microbiome community.

## Abstract (web summary)

Integration of differential and conventional RNA sequencing and transposon mutant fitness data for *Bacteroides thetaiotaomicron* grown under 15 different conditions provides an expression atlas, expands the regulatory RNA repertoire and reveals that the small RNA MasB regulates susceptibility to tetracyclines.

## Main

Bacteria of the Gram-negative *Bacteroides* genus are universal members of the gut microbiota of healthy human adults^1^. These bacteria occupy a hub position in the distal colon, influencing both host physiology and incoming enteric pathogens^2^, and serve as reservoirs of antibiotic resistance genes within the gastrointestinal tract^3^. Consequently, knowledge of the regulatory mechanisms underlying *Bacteroides* gene expression can help in the conception of microbiota-centric interventions to correct intestinal disorders.

Our current understanding of transcriptional control mechanisms in *Bacteroides* species mostly derives from studying their metabolic potential. Encoded on distinct clusters of neighbouring genes, these bacteria harbour numerous polysaccharide utilization loci (PULs)^4^, which allow them to feed on dietary fibre, as well as on host glycans^5^. PULs typically comprise regulatory systems—specific transcriptional regulators and sigma factors encoded within the same locus—that spur PUL transcription when the corresponding carbon source is sensed^6–10^. On a higher hierarchical level, a conserved global transcription regulator termed Cur^11^ coordinates *Bacteroides* carbohydrate utilization with other cellular processes^12,13^.

Complementing protein-mediated transcriptional control, bacteria universally employ small RNAs (sRNAs) that post-transcriptionally modulate gene expression via binding to complementary sequences within target messenger RNAs (mRNAs)^14^. While individual members of the *Bacteroides* genus are known to encode hundreds of sRNAs^15,16^, a primary bottleneck is that the vast majority of them do not yet have a known molecular function. Previously, we established Theta-Base^16^, a transcriptome database for *Bacteroides thetaiotaomicron*, which features the growth phase-dependent expression of sRNAs. However, these data were solely derived from experiments in nutrient-rich laboratory medium that falls short of reflecting in vivo-relevant conditions, composed of diverse stresses and nutritional variation. Besides, genome-wide phenotypic screens in *Bacteroides* species have so far been restricted to the analysis of mutations within coding genes^17–19^, yet knowledge of fitness-contributing noncoding genes could help to prioritize RNAs for functional studies in these health-relevant bacteria. To date, only few *Bacteroides* sRNAs have been partially characterized^15,16,20^, yet inactivation of none of them has been associated with a robust fitness phenotype, obscuring their importance for *Bacteroides*’ physiology.

In this Resource, we dissect global gene expression signatures in *B*. *thetaiotaomicron* type strain VPI-5482 under a range of host niche-related stresses and during growth on defined carbon sources. From the resulting transcriptomic compendium, we infer stress- and carbon source-specific gene expression patterns and identify noncoding RNAs. In an integrative approach, we use gene expression and mutant fitness data to link individual sRNAs to specific cellular processes. To demonstrate the value of our combined transcriptomics and functional genomics data, we focus on the previously uncharacterized sRNA MasB (BTnc201). Our findings assign MasB to the Cur regulon and suggest that this sRNA is a post-transcriptional regulator of a conserved tetratricopeptide protein, with phenotypic consequences when *B*. *thetaiotaomicron* is exposed to translation-blocking compounds.

## Results

### *B. thetaiotaomicron* transcriptome annotation

**Fig. 1:**
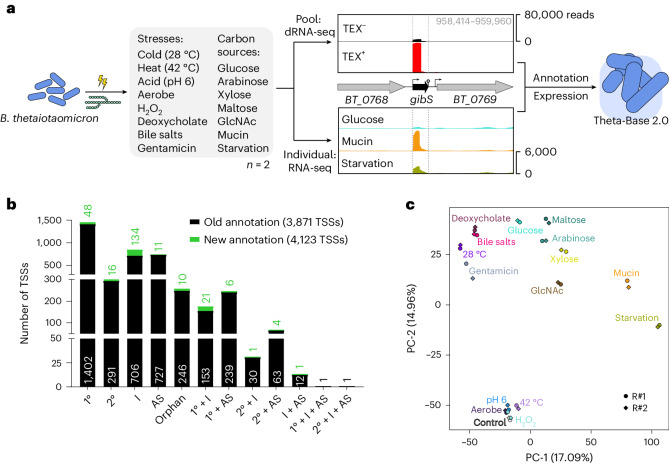
Comprehensive transcriptome annotation and gene expression profiling of *B*. *thetaiotaomicron*. **a**, Experimental outline. *B*. *thetaiotaomicron* type strain VPI-5482 was grown either in rich TYG medium to the mid-exponential phase (OD_600_ = 2.0) followed by a 2-h exposure to the indicated environmental stresses or in minimal medium supplemented with the indicated carbon sources to the late exponential phase (OD_600_ = 1.0) or—in case of mucin—early stationary phase (OD_600_ = 0.2). See Extended Data Fig. 1 for growth curves. RNA samples were either pooled for comprehensive TSS mapping via dRNA-seq or analysed separately via conventional RNA-seq. TEX, Terminator Exonuclease. **b**, Refined TSS annotations. See Methods for definitions of the different categories. 1°, primary; 2°, secondary; AS, antisense; I, internal. **c**, Principle component analysis of all of the RNA-seq samples analysed.

**Extended Data Fig. 1:**
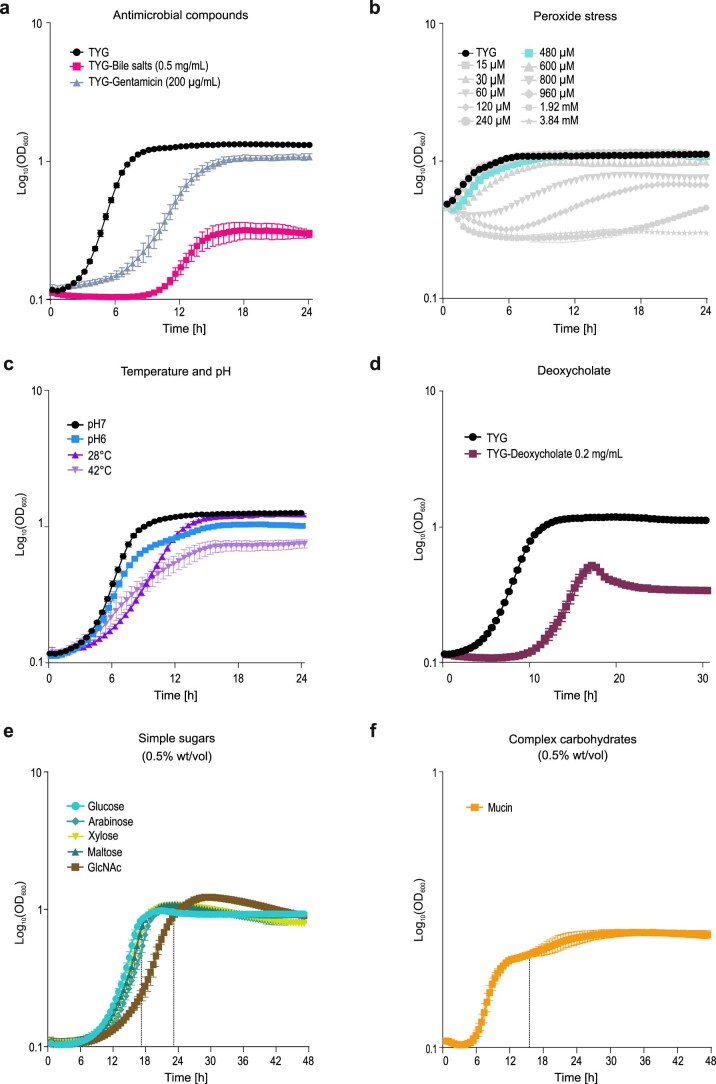
*B*. *thetaiotaomicron* growth curves over the profiled *in vitro* conditions. **a–d**, Growth in rich TYG medium upon constant exposure to the indicated environmental stresses. **e, f**, Growth in minimal medium with the indicated carbohydrates—simple sugars (**e**) and porcine mucin (**f**)—added as sole carbon sources. In each case, growth curves and error bars denote the mean ± SD from each three biological replicates. The dashed vertical lines in panels **e** and **f** denote the time points of sampling. Source data

To expand the transcriptome annotation of *B*. *thetaiotaomicron*, we compiled a suite of in vitro conditions that mimic specific aspects of this bacterium’s host niche (Fig. 1a and Supplementary Table 1). The large intestine exerts selective pressure on colonizing bacteria in the form of fluctuating pH levels, heterogeneous oxygen tension and the presence of secreted antimicrobial peptides and bile salts^21–24^. Consequently, our suite of stress conditions included moderate acidic pH, aerobic shaking, exposure to hydrogen peroxide, bile acids (deoxycholate or a bile salt mixture) and the antibiotic gentamicin (to which *Bacteroides* species are naturally resistant) and increased or decreased temperature (Extended Data Fig. 1a–d). To reflect metabolic fluctuations associated with the gastrointestinal tract, bacteria were grown in minimal medium supplemented with defined simple sugars (glucose, arabinose, xylose, maltose, *N*-acetyl-d-glucosamine (GlcNAc)) or porcine mucin glycans (Extended Data Fig. 1e,f), or they were nutrient deprived in minimal medium lacking a carbon source. Total RNA was extracted from the respective cultures and either pooled and analysed via differential RNA sequencing (dRNA-seq) for comprehensive transcription start site (TSS) mapping^25^ or sequenced separately via conventional RNA-seq to profile conditional gene expression. In all cases, library preparation was generic, resulting in the detection of both protein-coding and noncoding transcripts.

**Extended Data Fig. 2:**
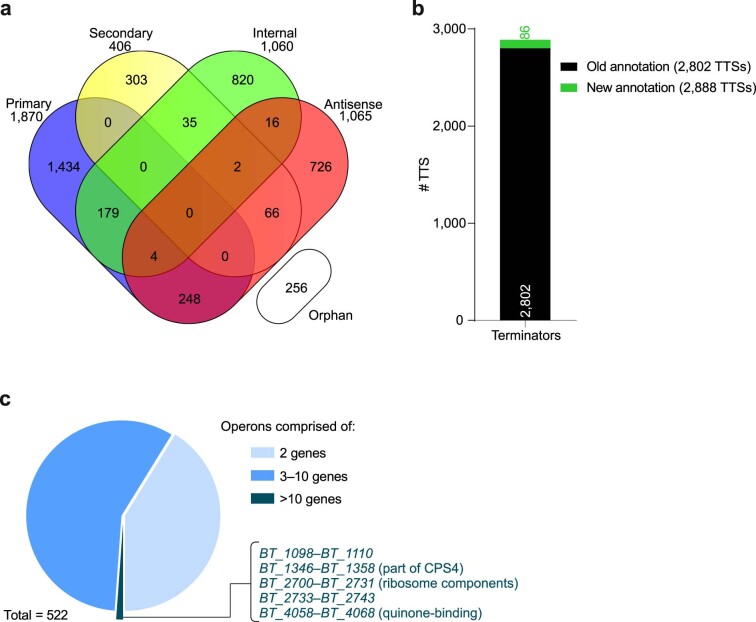
Updated transcription start site annotations, intrinsic terminator annotations and operon structures of *B*. *thetaiotaomicron*. **a**, Venn diagram showing the updated numbers of transcription start site categories. **b**, Refined transcription termination site (TTS) annotations. **c**, Operon structure prediction. Operons encompassing more than 10 genes are labeled by name.

The pooled complementary DNA (cDNA) sample was sequenced to ~40 million reads (that is, twice the depth that was previously considered to be sufficient to annotate the transcriptome of *Salmonella enterica* to saturation^26^). We analysed the resulting data using the ANNOgesic pipeline^27^ and collectively mapped the position of 4,123 TSSs across the *B*. *thetaiotaomicron* chromosome and plasmid. Comparing these results with our previously mapped TSSs, when *B*. *thetaiotaomicron* grew in rich TYG medium in the early or mid-exponential and stationary phase^16^, we found 252 unique TSS annotations contributed by the 15-condition pool (Fig. 1b, Extended Data Fig. 2a and Supplementary Table 2). Likewise, the number of transcription termination sites—predicted by a combination of read coverage drop and likelihood to fold into an intrinsic terminator hairpin (see Methods)—increased by 86 (Extended Data Fig. 2b). We also updated the annotation of operon structure predictions (Extended Data Fig. 2c). To better interpret *Bacteroides* transcriptomic features, we integrated our refined transcript boundary annotations with a map of invertible DNA regions (invertons) obtained by application of the PhaseFinder software^28^ to the *B*. *thetaiotaomicron* genome. Of the resulting 1,997 inverted repeats, 569 contained potential promoters (that is, involved sequences within a 50-base pair (bp) window upstream of a mapped TSS) and may represent sites contributing to *Bacteroides* phase-variable transcription initiation.

### *Bacteroides* stress response signatures

The conventional RNA-seq libraries were sequenced to 10–15 million reads per sample, as per general guidelines for bacterial differential expression analysis^29^. Of the 5,442 coding sequences in the *B*. *thetaiotaomicron* genome, 5,137 (94.4%) were expressed (more than ten reads per sample) under at least one of the 15 experimental conditions. Biological replicate samples clustered closely (Fig. 1c), indicating the absence of major batch effects. To aid in interpretation of the gene expression data, we compiled functional information by merging Kyoto Encyclopedia of Genes and Genomes (KEGG) and Gene Ontology term annotations with manually collated gene sets and regulons retrieved from literature research (see Methods and Supplementary Table 3).

**Fig. 2:**
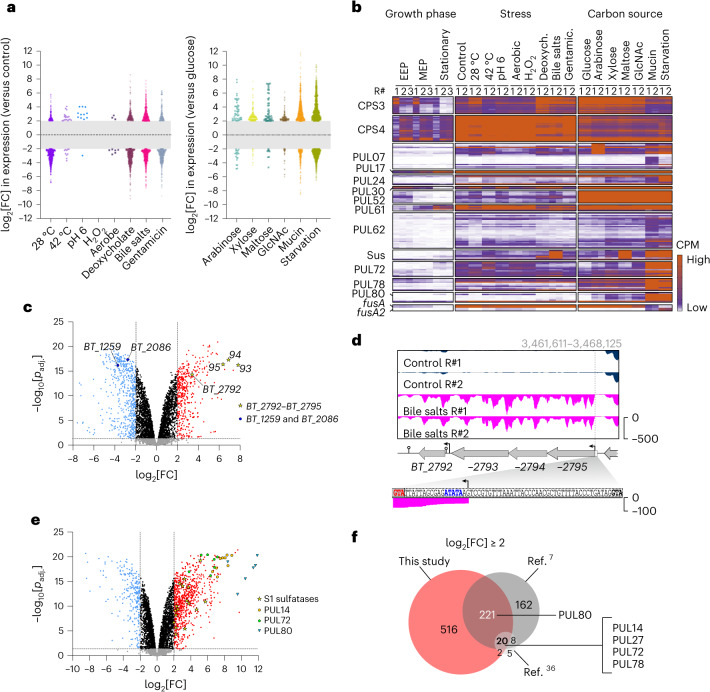
Stress response- and carbon source-specific expression of *B*. *thetaiotaomicron* genes. **a**, Bee swarm plots indicate global expression changes of *B*. *thetaiotaomicron* genes upon environmental shocks (left) or growth in the presence of the indicated sole carbon sources (right). Each dot denotes a bacterial gene that was up- (log_2_[FC] > 2; FDR < 0.05) or downregulated (log_2_[FC] < −2; FDR < 0.05) compared with the unstressed control condition in TYG medium, the growth in minimal medium plus glucose or, in case of starved bacteria, growth in rich TYG, respectively. The grey central bars delimit log_2_[FC] values that are not significant as per the above definition. **b**, Heat map showing the abundance of transcripts belonging to CPS3 or CPS4, or that are derived from selected PULs or from the elongation factor G-encoding mRNAs *BT_2729* (*fusA*) and *BT_2167* (*fusA2*). Growth phase-dependent expression data stem from ref. ^16^. Deoxych., deoxycholate; EEP, early exponential phase; gentamic., gentamicin; MEP, mid-exponential phase. The complete data for all eight CPSs and 96 PULs are provided in Extended Data Fig. 4. **c**, Volcano plot of differentially expressed genes when bacteria were exposed to bile salts compared with an unstressed control condition. Yellow stars denote genes of an operon coding for a bile salt tolerance-conveying efflux pump^19^ and blue circles label genes encoding bile acid-altering enzymes^68^. The generalized linear model likelihood ratio test implemented in edgeR was used to test for differential expression. *P*_adj._, adjusted *P* value. **d**, Re-annotation of the *BT_2792*–*BT_2795* operon was prompted by a previously undiscovered TSS downstream of its originally annotated start codon (bold black; proposed actual start codon in red; potential ribosome binding site^69^ in blue). The dashed line marks the position of the identified TSS (bent arrow symbol). **e**, Volcano plot showing gene expression changes as *B*. *thetaiotaomicron* feeds on mucin compared with glucose. S1 sulfatase genes^70^ are indicated by yellow stars and PUL14, −72 and −80 genes by orange circles, green pentagons and blue triangles, respectively. Differential expression was tested using edgeR’s generalized linear model likelihood ratio test. The dashed lines in **c** and **e** denote the thresholds for significantly regulated transcripts (under the criteria specified in **a**). **f**, Venn diagram displaying the overlap of mucin-specific gene induction between our dataset and those obtained in refs. ^7,36^. Source data

**Extended Data Fig. 3:**
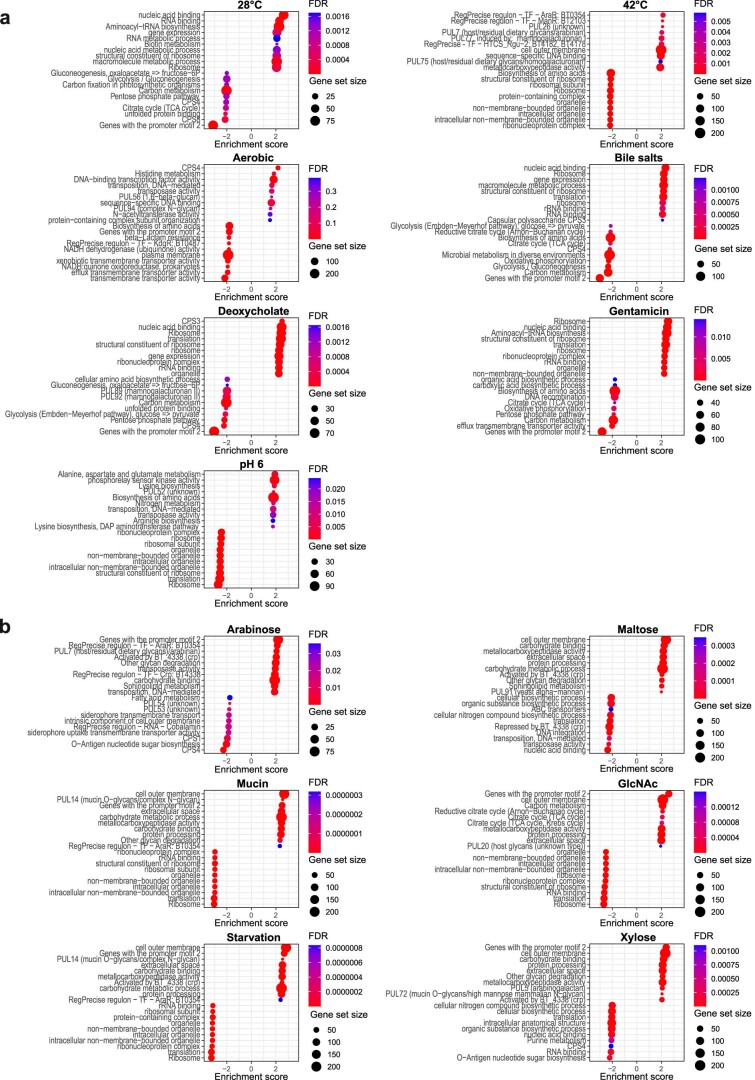
Gene set enrichment analyses. Top 10 enriched (normalized enrichment score >0) and depleted (normalized enrichment score <0) gene sets in the stress (**a**) and carbon source conditions (**b**). Gene sets derive from the custom gene set list described in the Methods section under ‘Gene set annotation and enrichment analyses’. Each gene set is represented by a circle whose size is proportional to the number of included genes (gene set size). Known inducers of PULs are included in brackets after the PUL name.

Pairwise comparisons of brief stress exposures to an unstressed control sample (Supplementary Table 4) and an ensuing gene set enrichment analysis (Extended Data Fig. 3a and Supplementary Table 5) revealed *Bacteroides* transcriptomic responses to environmental cues. Heat, mild acidic pH and aerobic exposure triggered only few, yet specific expression changes (Fig. 2a and Extended Data Fig. 5b–d), whereas substantial transcriptomic reprogramming was observed when bacteria faced cold, bile or sub-lethal antibiotic stress (Fig. 2a,c and Extended Data Fig. 5a,f). Brief exposure to hydrogen peroxide did not induce any significant expression changes, probably because the selected concentration (480 µM) was relatively low. Generally, stress-specific marker genes inferred from the literature showed the anticipated alterations (Supplementary Text). In the case of the *BT_2792*–*BT_2795* operon that encodes a bile salt tolerance-conveying efflux pump^19^, we observed a TSS and alternative start codon 60 nucleotides downstream of the annotated one (red and black ATG sequence in Fig. 2d). Since the previously annotated amino terminus was not supported by any sequencing reads (Fig. 2d, bottom), we re-annotated *BT_2795* accordingly.

**Extended Data Fig. 4:**
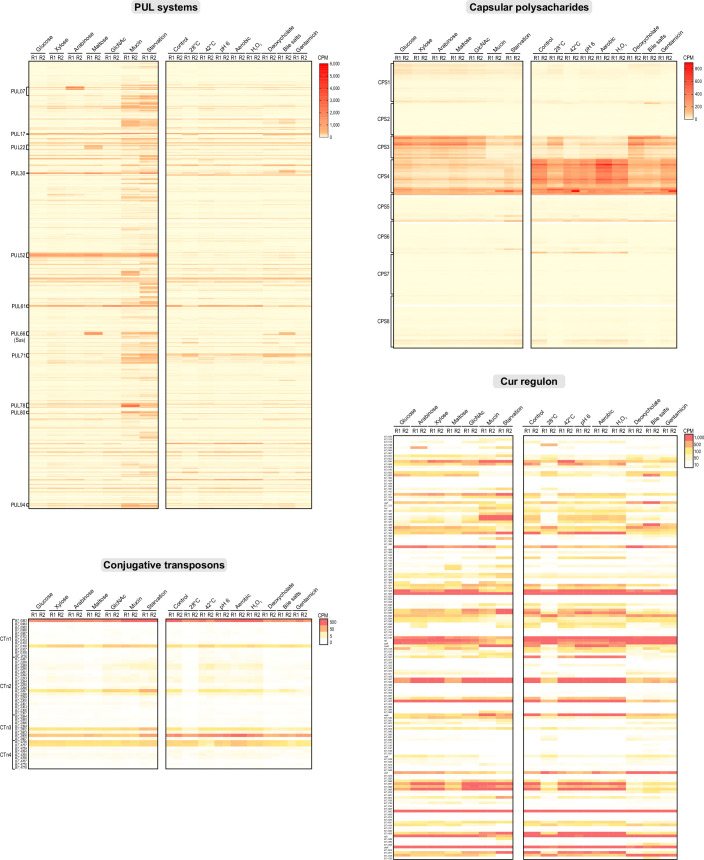
Transcript levels of *B*. *thetaiotaomicron* PUL systems, Cur regulon members, capsular polysaccharides, and conjugative transposons. For each condition and each replicate, the read counts per million (CPM) mapped to individual PUL genes (as inferred from PULDB^4^), to individual members of the Cur regulon as inferred from (Supplementary Table S3B in Ref. ^13^), to CPS genes, and to CTn loci are plotted. Selected, strongly affected PULs and CPSs are labeled and re-plotted in Fig. 2b.

*Bacteroides* genomes harbour multiple capsular polysaccharide (CPS) loci, allowing these bacteria to alter their surface structure with consequences for the evasion of host immunity and phage attack^30^. Invertible promoters—included in our inverton map—result in phase-variable expression of certain CPS loci^31^. Of the eight CPSs of *B*. *thetaiotaomicron*, CPS4 and, to lesser extent, CPS3 were dominant during in vitro growth (Extended Data Fig. 4), recapitulating previous findings^32^. The relative CPS dominance changed (from CPS4 to CPS3) when bacteria were exposed to the secondary bile acid deoxycholate (Fig. 2b and Extended Data Fig. 6a). Exposure to a bile salt mixture, gentamicin or the cold led to a partial induction of CPS3. Interestingly, the updated Theta-Base annotation features four predicted *cps3* sub-operons, only the first of which was upregulated under these conditions (operon 65 in Extended Data Fig. 6b), which we interpret as a snapshot during a gradual shift from CPS4 to CPS3 expression—as opposed to an outright CPS switch—during certain stresses.

### Carbon source-specific gene expression patterns

**Extended Data Fig. 5:**
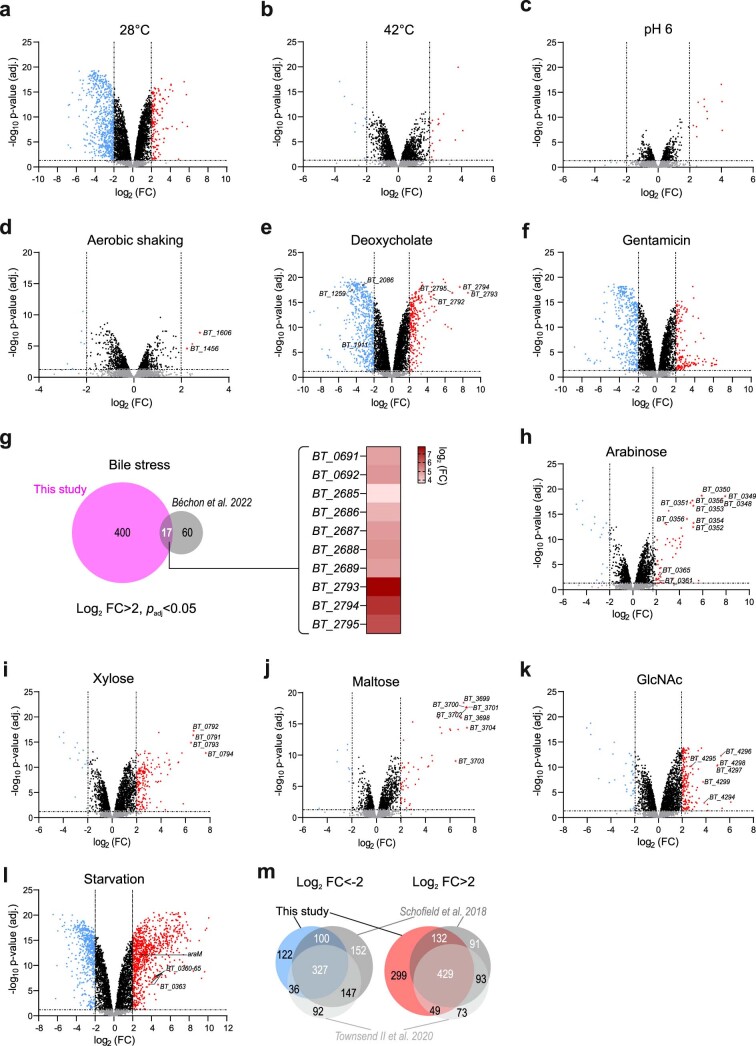
Pair-wise comparison of stress- and carbon source-specific gene expression changes. **a–f**, Volcano plots report differential expression of *B*. *thetaiotaomicron* exposed to the indicated stresses (x-axis) over significance (y-axis). The generalized linear model likelihood ratio test implemented in edgeR was used to test for differential expression. **a**, Cold vs. TYG control. **b**, Heat vs. TYG control. **c**, Acidic vs. TYG control. **d**, Aerobic shaking vs. TYG control. The genes for cytochrome C peroxidase (*BT_1606*) and thioredoxin (*BT_1456*) are labeled. **e**, Deoxycholate vs. TYG control. Genes belonging to the *BT_2792*-*BT_2795* operon^19^ or encoding bile acid-altering enzymes^68^ are labeled. **f**, Gentamicin vs. TYG control. **g**, Venn diagrams display the overlap of bile salts-specific gene expression (significantly upregulated) between our dataset and that obtained in Ref. ^71^. The associated heat map depicts highly upregulated operons including components of two efflux systems (*BT_2793*-*BT_2795*, *BT_2685*-*BT_2689*) as well as an outer membrane protein and a calcineurin superfamily phosphohydrolase (*BT_0691*-*BT_0692*). **h–l**, Volcano plots report differential expression of *B*. *thetaiotaomicron* feeding on the indicated carbon sources (x-axis) over significance (y-axis). **h**, Arabinose vs. glucose. Genes belonging to the arabinose utilization operon *BT_0348*-*BT_0369* (Ref. ^12^) are indicated. **i**, Xylose vs. glucose. Genes belonging to the xylose utilization operon *BT_0791*-*BT_0794* (Ref. ^13^) are indicated. **j**, Maltose vs. glucose. Genes belonging to the starch utilization system (*sus*) operon *BT_3704*-*BT_3698* (Refs. ^72,73^) are labeled. **k**, *N*-acetyl-D-glucosamine vs. glucose. PUL80 genes are labeled. **l**, Starvation vs. TYG. The genes for the aldose 1-epimerase precursor AraM and for arabinose-utilizing PUL07 are marked. **m**, Venn diagram denotes the overlap of starvation-induced up- or downregulations observed here and in previous studies^13,74^. Source data

To dissect *B*. *thetaiotaomicron* metabolic programs, we calculated differential gene expression upon growth in the different carbon sources relative to cultures feeding on glucose (Extended Data Fig. 5 and Supplementary Table 4) and again determined enriched gene sets (Extended Data Fig. 3b and Supplementary Table 5). The number of significantly differentially expressed genes (log_2_[fold change (FC)] < −2 or > +2 and false discovery rate (FDR) < 0.05) tended to increase with the complexity of the carbon sources (Fig. 2a). Generally, PUL expression responded to known substrates, adding further confidence to our dataset (Fig. 2b and Supplementary Text).

**Extended Data Fig. 6:**
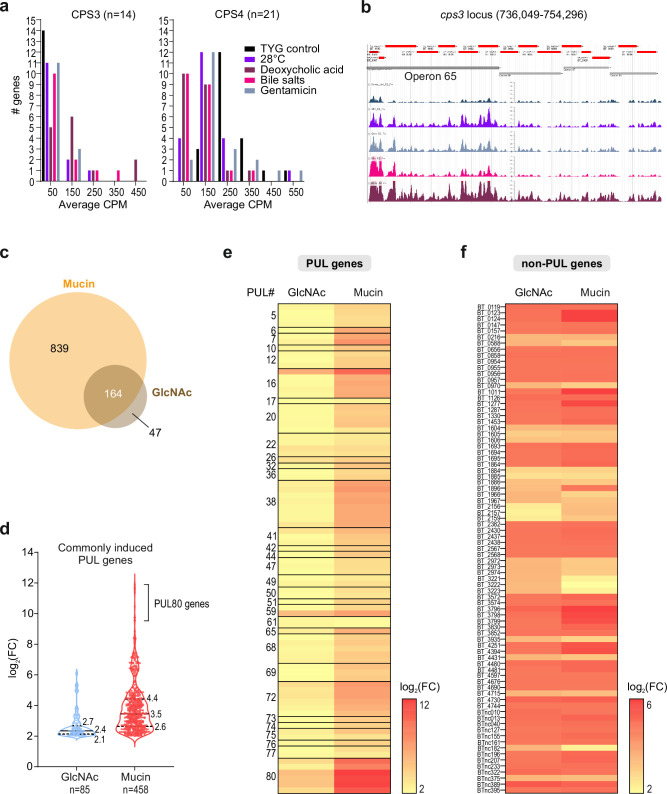
A shift in CPS expression and the overlap between GlcNAc- and mucin-responsive genes. **a**, Expression of CPS3 (left) and CPS4 (right) genes across selected stress conditions, namely 28 °C, deoxycholate, bile salts and gentamicin in comparison to the TYG control. Genes were grouped into bins (width=100) based on average CPM values per condition. **b**, Theta-Base screenshot of the RNA-seq read coverages across the *cps3* locus. Sub-operon #65, to which the main text refers to, is boxed. Color-coding is the same as for panel a. **c**, Venn diagram denotes the overlap in the regulated genes between *N*-acetyl-D-glucosamine- and mucin-consuming bacteria. **d**, The violin plots depict the fold-change in expression of individual PUL-associated genes induced in the presence of GlcNAc (*n* = 85) and polymeric mucin (*n* = 458) relative to the reference sugar glucose. Each dot refers to a single gene and the solid and dashed lines indicate the median and quartiles, respectively. **e, f**, Expression profiles of commonly induced PUL genes (*n* = 83) (**e**) and of non-PUL genes (*n* = 81) (**f**) in the presence of GlcNAc or mucin relative to that of glucose. Source data

When polymeric mucin was the sole carbon source, the high mannose mammalian *N*-glycan utilization system PUL72 (average log_2_[FC] = 5; ref. ^33^) and host-derived mucin *O*-glycan-processing systems PUL14, PUL78 and PUL80 (mean log_2_[FC] = 7, 5.2 and 10.5, respectively^7,34,35^) were strongly induced (Fig. 2b,e). PUL62, whose inducer is currently not known, was also upregulated (average log_2_[FC] = 2.5; Fig. 2b), suggesting that this PUL responds to mucin-derived glycans. Overall, a substantial fraction of the mucin-activated genes identified here overlapped with genes previously found to be upregulated in *B*. *thetaiotaomicron* growing in vitro on a glycan mixture prepared from the porcine gastric mucosa^7^ or colonizing the outer mucous layer of C57BL/6 mice^36^ (Fig. 2f and Supplementary Table 6). In addition, we noticed an overlap between the set of mucin-regulated genes and genes differentially expressed in the presence of its constituent monosaccharide, GlcNAc (Extended Data Fig. 6c,d). Induction of the 83 common PUL-associated genes was generally more pronounced in mucin than in GlcNAc, whereas expression of the 81 common non-PUL genes was at rather similar levels (Extended Data Fig. 6e,f and Supplementary Table 7). This would be in line with a model wherein GlcNAc is less repressive towards basal mucin PUL expression than other, unrelated simple sugars, leading to comparably small PUL expression changes during growth on GlcNAc, while growth on polymeric mucin induces larger changes in specific PULs.

Inspection of known members of the regulon governed by the transcriptional master regulator of carbohydrate utilization, Cur^13^, indicated major gene expression changes, particularly when bacteria consumed mucin or were starved (Extended Data Figs. 4 and 5l,m), in accordance with previous reports^12,13^. For instance, expression of *fusA2* (*BT_2167*), which is an established Cur target^13^ and encodes the alternative translation elongation factor G2 (EF-G2), was induced in mucin and peaked during carbon deprivation (Fig. 2b). The inverse expression pattern was observed for *fusA* (*BT_2729*) (Fig. 2b), encoding the canonical EF-G and not belonging to the Cur regulon^13^. This corroborates previous reports^12,13,37^ and further supports that *B*. *thetaiotaomicron* utilizes a distinct protein synthesis machinery during colonization of a sugar-deprived host niche. In summary, the combined data corroborate former reports, but also extend our collective knowledge of conditional gene expression in *B*. *thetaiotaomicron*.

### Conditional expression of noncoding genes

**Fig. 3:**
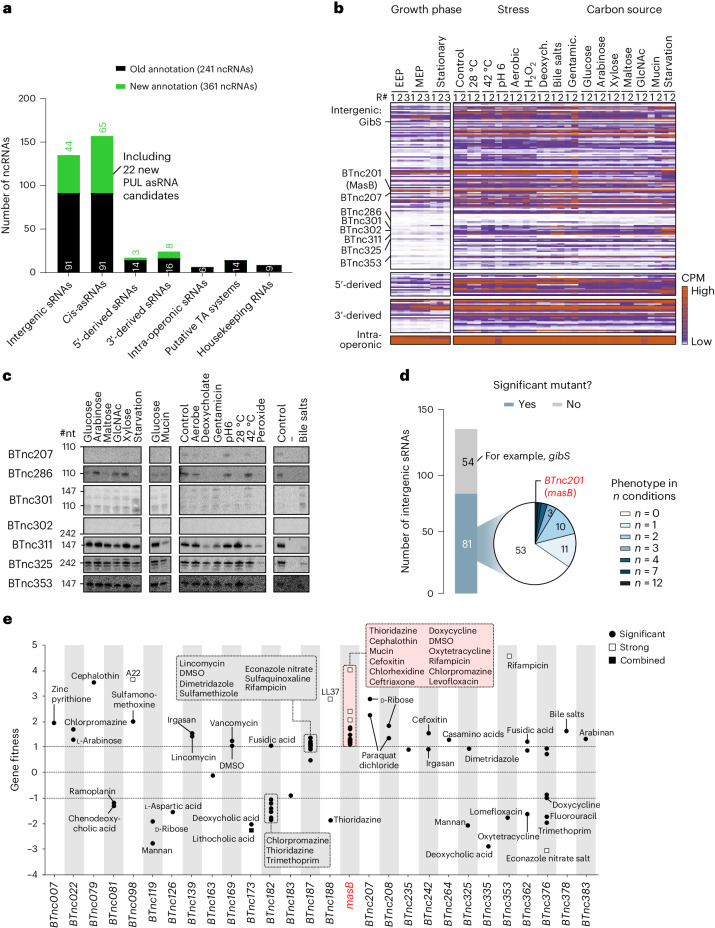
*Bacteroides* fitness-influencing sRNAs. **a**, Identification of noncoding RNA (ncRNA) candidates in *B*. *thetaiotaomicron*. For each class, the numbers of previously annotated (ref. ^16^; over growth in rich medium; black) and here-identified candidates (green) are plotted. TA, toxin-antitoxin. **b**, Heat map showing the conditional expression of the different classes of sRNAs. Growth phase-dependent expression data stem from ref. ^16^. **c**, Northern blot-based validation of the existence and expression profile of previously undescribed sRNA candidates. The depicted blots are representative of two biological replicates. #nt, number of nucleotides. **d**,**e**, Fitness data of *B*. *thetaiotaomicron* sRNA mutants, obtained by reanalysing the dataset from ref. ^19^. The sRNAs are grouped based on the number of significant fitness changes associated with their transposon disruption (**d**) and, for sRNA mutants with at least one phenotype, the respective condition(s) and fitness score(s) are plotted (**e**). The categories in **e** (that is, combined, significant and strong) are defined in the Methods. *BTnc201* (*masB*) and *masB* in **d** and **e** refer to the gene encoding the MasB sRNA (formerly BTnc201). DMSO, dimethyl sulfoxide. Source data

Unlike the situation for *B*. *thetaiotaomicron* protein-coding genes, there is hardly any information in the literature with respect to stress- and metabolism-related expression of noncoding genes of this bacterium. Interestingly, and in contrast with the relatively small fraction of additional TSSs and transcription termination sites gained from the pooled dRNA-seq experiment (Fig. 1b and Extended Data Fig. 2b), the extended dataset resulted in a substantial increase in the number of *B*. *thetaiotaomicron* noncoding RNA candidates (Fig. 3a). For example, following manual curation (see Methods), we confidently predict 135 intergenic sRNAs, 44 of which were identified in this study.

**Extended Data Fig. 7:**
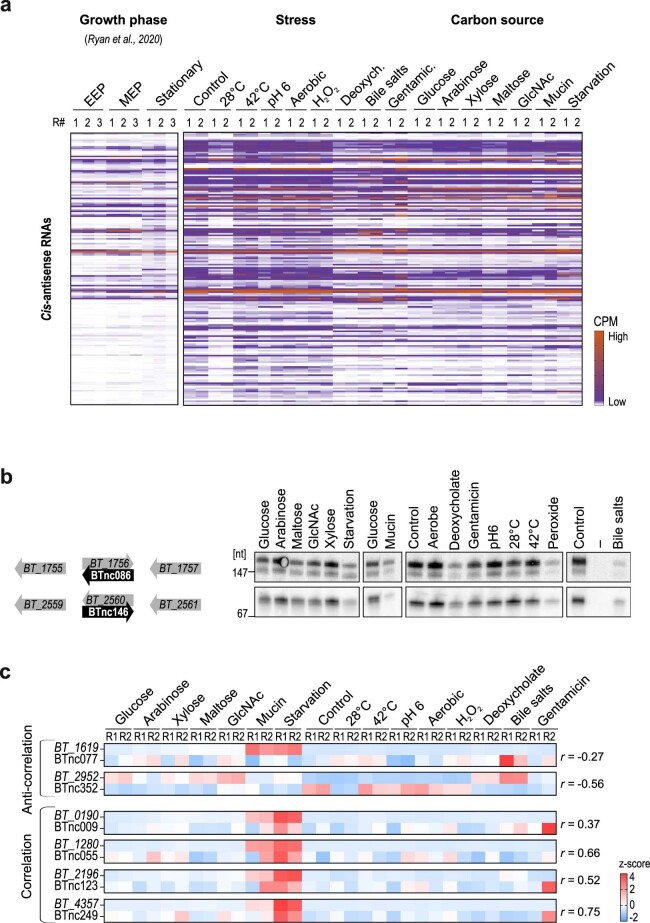
Expression of *B*. *thetaiotaomicron cis*-encoded antisense RNAs. **a**, Heat map showing the expression of annotated *cis*-asRNAs across the set of experimental conditions. Growth phase-dependent expression data stem from Ref. ^16^. EEP, early exponential phase; MEP, mid-exponential phase; CPM, counts per million. **b**, Northern blot-based validation of newly predicted asRNA candidates. Shown are representative northern blots of two biological replicates. **c**, The heat map shows *z*-scores of log_2_FC values of PUL-associated antisense RNAs and their respectively overlapping PUL gene (*susC* homologue). This reveals patterns of correlation and anti-correlation, as indicated by Pearson’s *r* values at the right. Source data

Generally, the basal abundance levels of sRNAs varied substantially and we observed several differentially expressed candidates across our conditional dataset (Fig. 3b and Extended Data Fig. 7a). Expression of the established sRNA GibS, for example, was upregulated in GlcNAc (as previously reported^16^), but peaked when bacteria fed on mucin or were starved. Using northern blotting, we validated the expression of seven of the here-predicted sRNAs (Fig. 3c). This included the acid-induced expression of BTnc207, the starvation-specific accumulation of BTnc302 and the downregulation of BTnc311 and BTnc325 during bile stress.

A previous study discovered a family of antisense RNAs (asRNAs) divergently encoded to PUL operons^15^. The observed anti-correlation in expression between several of these antisense–sense pairs suggested that asRNAs repress their cognate PUL—a mechanism validated exemplarily for one PUL-associated asRNA in *Bacteroides fragilis*^15^. In *B*. *thetaiotaomicron*, we recently annotated ten PUL-associated asRNAs^16^; the present data further increased this number to 32 (Fig. 3a) and we validated two of the predicted candidates by northern blotting (Extended Data Fig. 7b). What is more, the comprehensive metabolic expression data now allowed us to probe this anti-correlation phenomenon on a more global scale. We again found examples where an asRNA’s expression inversely mirrored that of its cognate PUL operon, but also observed counter-examples of positive correlation in individual asRNA–PUL pairs (Extended Data Fig. 7c). In other words, the extended transcriptomic data revealed a more nuanced picture of PUL-associated asRNAs than was anticipated and further enhance the need for functional characterization of this specialized class of noncoding RNAs.

### Phenotypes associated with *Bacteroides* sRNA inactivation

**Extended Data Fig. 8:**
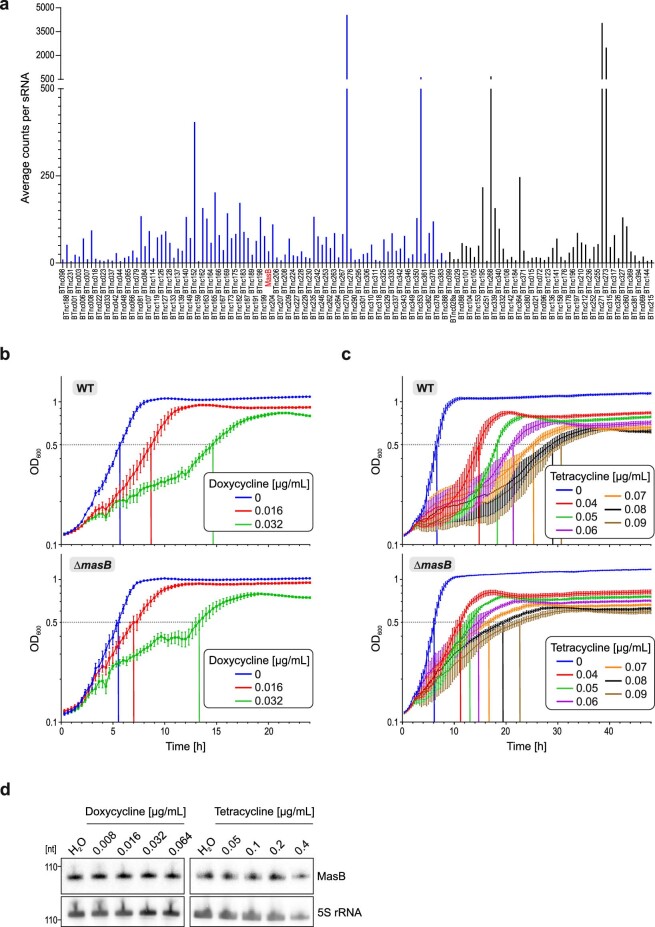
TIS screen for phenotypes associated with *B*. *thetaiotaomicron* sRNAs and role of MasB in antibiotic tolerance. **a**, Average number of transposon insertions per ncRNA gene in the mutant library across the range of different sample conditions. Intergenic *B*. *thetaiotaomicron* sRNAs are shown in blue, and 5′- or 3′-derived, or intra-operonic sRNA candidates in black. In this study, we focused on only the intergenic sRNA mutants. **b, c**, Growth curves of *B*. *thetaiotaomicron* isogenic wild-type (upper) and ∆*masB* (lower) in TYG supplemented with increasing concentrations of doxycycline (**b**) or tetracycline (**c**). Plotted are the means ±SD from each three biological replicate experiments, that each comprised technical duplicates. Indicated with dotted lines are the times to reach an OD_600_ of 0.5 for each strain and treatment. **d**, Antibiotics exposure does not majorly influence MasB steady-state levels. Northern blot on total RNA samples derived from *B*. *thetaiotaomicron* cultures exposed for 2 h to increasing concentrations of either doxycycline or tetracycline, relative to RNA from vehicle (water)-treated control cultures. 5S rRNA was the loading control. Shown are representative northern blots of two biological replicates. Source data

To provide support for the involvement of individual noncoding RNAs in specific cellular processes, we reanalysed an existing high-throughput transposon insertion sequencing (TIS) dataset from *B*. *thetaiotaomicron* grown under 490 defined experimental conditions^19^. For practical reasons, we focus here on standalone sRNA genes, encoded as independent transcriptional units and not overlapping with other genetic features. Of these 135 intergenic sRNAs, 81 were represented in the transposon mutant library (Fig. 3d and Extended Data Fig. 8a). Mutants of 28 sRNAs exhibited a statistically significant fitness change (|*t*| > 4) compared with the other mutants in the pool in at least one successful experiment (as defined in ref. ^19^; that is, an experiment in which a gene is represented by a sufficient number of barcode counts) (Fig. 3d,e and Supplementary Table 8). The majority of intergenic sRNAs affecting fitness showed a condition-specific phenotype when disrupted (Fig. 3d). However, in the case of a handful of sRNAs, disruption resulted in broader competitive fitness changes. In the following, we focused on the MasB sRNA, whose inactivation led to the highest predicted number of significant fitness phenotypes among all intergenic sRNA mutants.

### MasB confers antibiotics susceptibility

**Fig. 4:**
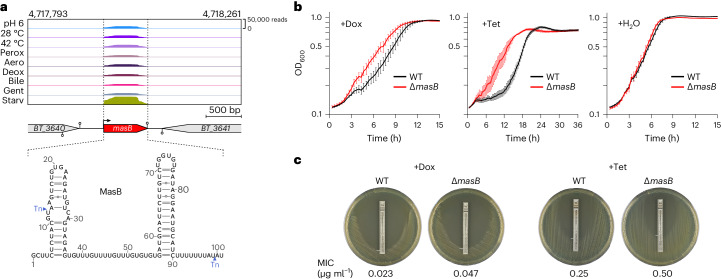
Genetic depletion of *masB* confers *B*. *thetaiotaomicron* enhanced tolerance of doxycycline and tetracycline. **a**, Top: MasB expression in differently stressed *B*. *thetaiotaomicron* cultures. Shown are representative read coverages across the *masB* genomic locus and flanking genes. Bottom: experimentally determined secondary structure of MasB^38^. The positions of the inserted transposons are indicated by blue arrowheads. The insertion at position 12/13 results in a functional *masB* null mutant, while the insertion at position 99/100 is unlikely to affect this sRNA’s activity. Aero, aerobe; Bile, bile salts; Deox, deoxycholate; Gent, gentamicin; Perox, peroxide; Starv, starvation. **b**, Growth curves of a *B*. *thetaiotaomicron* ∆*masB* mutant (red) and its isogenic wild-type (black) in TYG, supplemented with either 0.016 µg ml^−1^ doxycycline (+Dox; left), 0.05 µg ml^−1^ tetracycline (+Tet; middle) or water as the vehicle control (+H_2_O; right). Plotted are the means ± s.d. from three biological replicate experiments, each comprising technical duplicates. **c**, Antibiotic susceptibility testing to determine the minimal inhibitory concentration (MIC) of the indicated strains in response to doxycycline and tetracycline, which is defined as the point on the antibiotic strip that is intersected by the growth inhibition ellipse. Representative images from five (doxycycline) or three (tetracycline) biological replicate experiments are depicted. Source data

MasB (previously BTnc201; renamed here for reasons to follow) is a roughly 100-nucleotide-long, narrowly conserved sRNA^38^. Relative to the transcripts from its flanking genes, MasB accumulated to high steady-state levels under all of the experimental conditions tested here, but peaked when bacteria were starved (Fig. 4a). The TIS analysis suggested that *masB* disruption promotes growth upon exposure to diverse antibiotics and antimicrobials (Fig. 3e). This included enhanced fitness of bacteria with mutated *masB* during exposure to tetracycline derivatives (oxytetracycline and doxycycline hyclate). We confirmed this phenotype using a clean deletion mutant of this sRNA and increasing concentrations of doxycycline, both during growth in liquid medium (Fig. 4b and Extended Data Fig. 8b) and on solid agar (Fig. 4c). Similar effects were observed when exposing the strains to conventional tetracycline (Fig. 4b,c and Extended Data Fig. 8c). MasB expression did not respond to antibiotic exposure (Extended Data Fig. 8d), yet the associated fitness effects were stress specific, as the mutant grew indistinguishably from an isogenic wild-type strain in vehicle-treated control cultures (Fig. 4b). Based on these results, we concluded that MasB confers *Bacteroides* sensitivity to ribosome-targeting antibiotics of the tetracycline family. We hence name this sRNA MasB, for modulator of antibiotics susceptibility in *Bacteroides*.

### Assignment of MasB to the Cur regulon

**Fig. 5:**
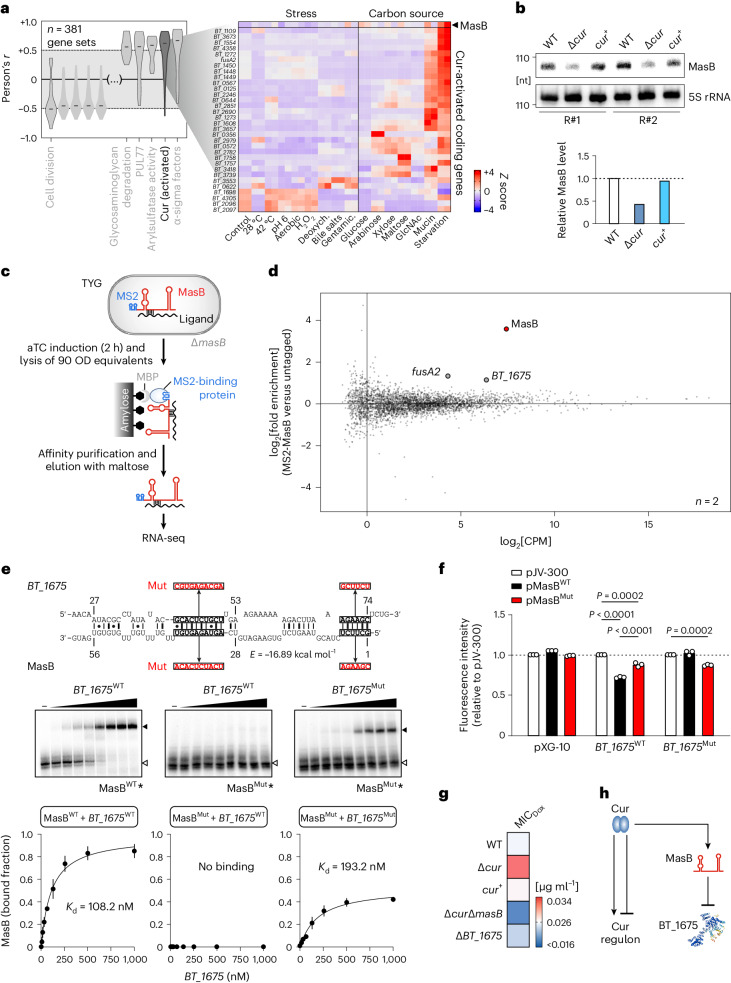
MasB is assigned to the Cur regulon and targets *BT_1675* mRNA. **a**, Left: Pearson’s correlation coefficients between MasB expression and that of annotated *Bacteroides* gene sets. Gene sets with |median *r*| > 0.5 are labelled. Right: expression of MasB and Cur-induced targets across the set of experimental conditions. **b**, Top: RNA was collected from cultures upon 2 h of carbon starvation and analysed by northern blot. Bottom: quantification of MasB band intensity relative to 5S rRNA, with the wild-type ratio set to one, over the two biological replicates. **c**, Schematic of the MAPS workflow. MBP, maltose-binding protein. **d**, Mean difference plot showing the log_2_[FC] and average abundance of each gene in MS2-MasB versus the untagged control, merged over biological duplicates. **e**, Top: depiction of the top-ranked in silico-predicted interaction region between MasB and *BT_1675* mRNA. The numbers refer to nucleotide positions relative to the translational start codon (in the case of the mRNA) or TSS (for MasB). Middle: in vitro-transcribed and radio-labelled wild-type or mutant MasB RNA was incubated with increasing concentrations of a 137-nucleotide-long 5′ segment of *BT_1675* mRNA or the correspondingly mutated mRNA segment. Black and white arrowheads indicate free and bound MasB, respectively. Bottom: dissociation curves showing the mean ± s.d. from three technical EMSA replicates. Mut refers to defined base pair-exchange mutants designed to disrupt or restore binding between MasB and its predicted target mRNA, respectively. **f**, GFP reporter assays. The 5′ region of wild-type or mutated *BT_1675* (Mut; same mutations as depicted in **e**) was fused to GFP and the resulting construct transformed into *E*. *coli* TOP10. A MasB expression plasmid (WT or Mut) or a plasmid harbouring a nonsense sequence (pJV-300) was co-transformed and the respective strains were cultured overnight in Lysogeny Broth medium, before flow cytometry was performed. The control plasmid pXG-10 harbours only the GFP cassette. The bars denote the means of three biological replicates. For significant comparisons, *P* values are given (one-way ANOVA with Tukey’s multiple comparisons test). The horizontal dashed lines in **b** and **f** denote levels in the respective controls, set to 1. **g**, MIC of doxycycline for the indicated strains. Means from eight biological replicate experiments are plotted (see Supplementary Table 9 for raw data and Extended Data Fig. 10d for growth curves of the corresponding strains). **h**, Schematic of the MasB regulatory axis. Source data

Our transcriptomics dataset lends itself for co-expression analysis to obtain insight into cellular regulatory circuits. Here, as an illustrative use case, we performed co-expression analysis for MasB (Fig. 5a), which grouped the sRNA among genes that are activated by Cur—the transcriptional master regulator of carbohydrate utilization in *B*. *thetaiotaomicon*^12,13^. This prompted us to explore the hypothesis that transcription of MasB might also be governed by Cur. Indeed, closer inspection of publicly available chromatin immunoprecipitation and sequencing data revealed that one of the most significant Cur binding sites in the *B*. *thetaiotaomicron* chromosome is located upstream of the *masB* gene (peak ID #598 in ref. ^13^). To assess the impact of this transcription factor on MasB expression, we deleted *cur* from the chromosome. During carbon source deprivation (that is, the condition when Cur activation is maximal (Fig. 5a)), the level of MasB was more than twofold decreased in the ∆*cur* strain compared with the wild-type strain (Fig. 5b). This effect could be complemented in *trans*. We conclude from these experiments that Cur acts as a transcriptional activator of the MasB sRNA.

### MAPS predicts *BT_1675* as a direct MasB target

**Extended Data Fig. 9:**
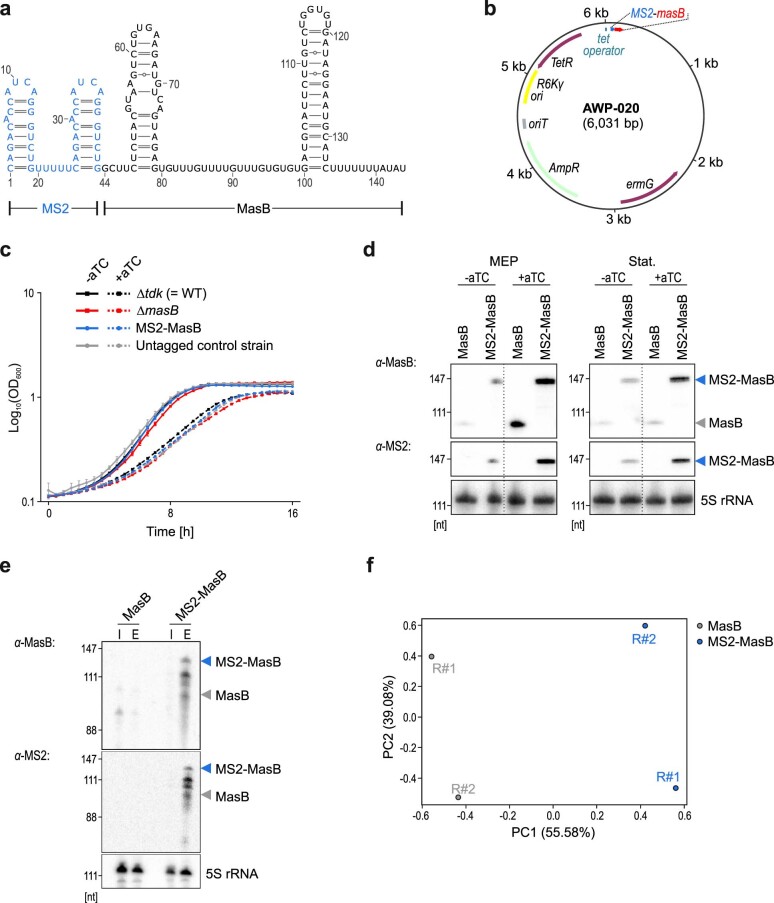
Establishment of MAPS for *B*. *thetaiotaomicron* sRNA MasB. **a**, *In*-*silico* prediction of the secondary structure of a fusion of the 5′ end of MasB to the MS2 aptamer using the RNAfold WebServer^75^. **b**, Plasmid map of AWP-020 containing the anhydrotetracycline-inducible MS2-MasB construct. **c**, Growth curves of the indicated strains in TYG medium in the absence (solid lines) or presence (dotted lines) of anhydrotetracycline (aTC; 200 ng/mL) as an inducer of MasB expression. Plotted values are the means of three biological replicates with error bars indicating the standard deviation. **d**, Northern blot to probe MS2-MasB in the strains used for MAPS grown in TYG to mid-exponential phase (MEP, ~7 h) or stationary phase (~10 h) in the absence or presence of anhydrotetracycline induction (for 2 h). The blot is representative of two biological replicates. **e**, Northern blot of the input (I) and eluate (E) fractions of the MAPS experiment, probed for MasB or MS2. 5S rRNA served as the loading control. An enrichment of MS2-MasB in the eluate compared to the input and to the samples derived from the untagged control strain demonstrates efficient capture and pull-down of MasB. The blot is representative of two biological replicates. **f**, Principal component analysis plot of the sequencing data revealed a segregation between the two biological replicates derived from the pull-down of MS2-MasB (blue) and those from the control strain (grey). Source data

**Extended Data Fig. 10:**
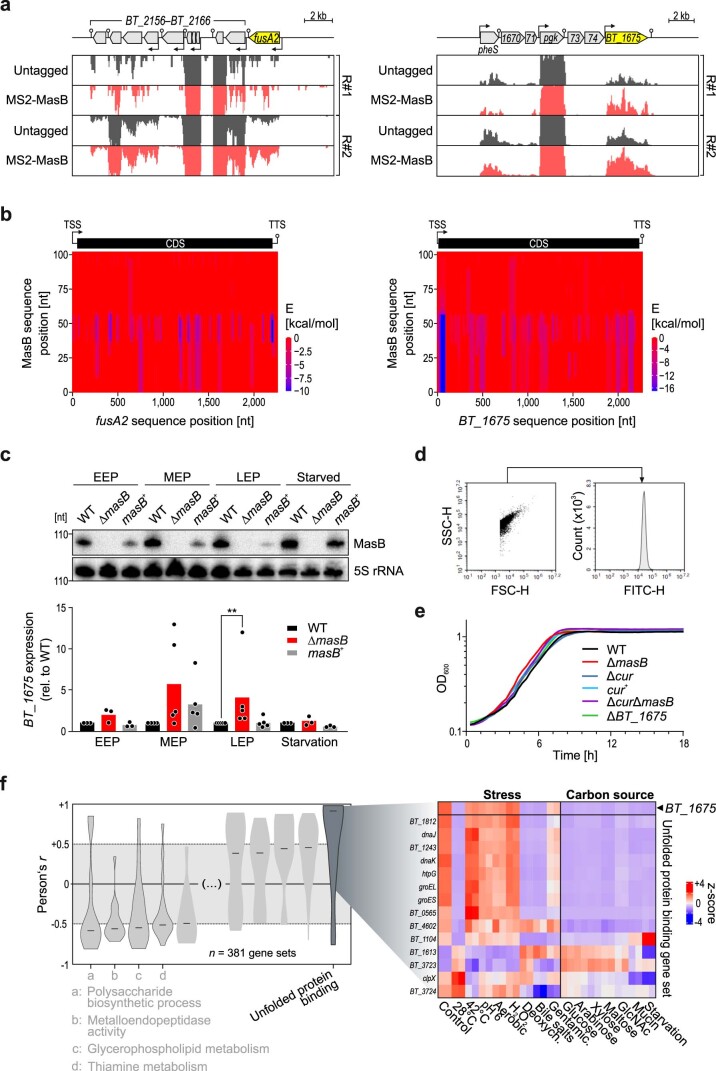
Characterization of the MasB target *BT_1675*. **a**, MAPS coverage plots across the *fusA2* and *BT_1675* loci. R#1 and #2 are biological replicates. **b**, *In*-*silico* prediction of putative MasB interaction sites within MAPS-derived target candidates. The heat maps display the position-wise minimal energy (E) profiles of *fusA2* or *BT_1675* mRNAs (x-axes), respectively, with MasB (y-axis) as retrieved from IntaRNA^41^. Full-length sequences from TSS to TTS have been queried and the positions are relative to the TSS in each case. **c**, The indicated strains were grown in TYG to early (EEP), mid- (MEP), or late exponential phase (LEP), and starved for 2 h in minimal media devoid of a carbon source. Total RNA samples were collected and analyzed via northern blotting (upper) and qRT-PCR (lower; bars denote the means of five biological replicates). 5S rRNA or 16S rRNA served as loading control or reference gene, respectively. ‘**’ refers to a *p*-value of 0.0022 (non-parametric t-test; Mann-Whitney test); all other comparisons were statistically insignificant (*p* ≥ 0.05). **d**, Graphical illustration of the gating strategy applied to the fluorescent reporter assay, whose results are plotted in Fig. 5f. Left: gating for intact bacterial cells in the forward/sideward scatter. Right: fluorescence intensity histogram (detected in the FITC channel). **e**, Growth curves of *B*. *thetaiotaomicron* mutant strains in TYG. The graphs refer to the means of each three biological replicates. **f**, Coexpression analysis of *BT_1675*. Left: violin plots of Pearson’s correlation coefficients between *BT_1675* expression and that of annotated *Bacteroides* gene sets (≥10 individual transcriptional units) sorted from left (lowest *r*) to right (highest *r*). Out of all 381 gene sets included in this analysis, the 5 top positively and negatively correlated (highest and lowest median *r*, respectively) are shown. Gene sets with |median *r*| > 0.5 are named below the plot. The GO term ‘Unfolded protein binding’ ranked first in absolute correlation. Right: heat map showing the expression of *BT_1675* and ‘Unfolded protein binding’ genes across the set of 15 different experimental conditions. Source data

To search for MasB targets, we established MS2 affinity purification and sequencing (MAPS) technology^39^ in *B*. *thetaiotaomicron* (Fig. 5c, Extended Data Fig. 9 and Supplementary Text). The top-enriched transcripts in the MS2-MasB co-purifications relative to the untagged background control were *BT_1675*, encoding a conserved tetratricopeptide domain protein, and the *fusA2* mRNA that encodes the alternative ribosomal factor EF-G2 (Fig. 5d and Extended Data Fig. 10a). To narrow in on potential targeting regions, we applied in silico prediction of partially complementary sequences between the sRNA and its presumed targets using the IntaRNA algorithm^40,41^. For *fusA2*, no convincing binding site was found; however, in silico prediction suggested that the *BT_1675* mRNA might be targeted ~40 nucleotides downstream of its translation start codon (Extended Data Fig. 10b). Electrophoretic mobility shift assays (EMSAs) confirmed MasB binding to the 5′ region of *BT_1675* mRNA in vitro (Fig. 5e). Notably, sequence mutations in the predicted interaction sites of MasB (positions 41–50 and 69–74 relative to the TSS) abrogated target binding, yet the interaction was partially restored with a compensatory mutant of *BT_1675* (Fig. 5e).

To test for an effect of MasB on the steady-state levels of *BT_1675* mRNA, we grew *B*. *thetaiotaomicron* wild-type, ∆*masB* and *masB*^+^ (a corresponding *trans*-complementation strain) cultures in TYG or starved them for 2 h in minimal medium. We then extracted total RNA and subjected the samples to northern blot and quantitative reverse-transcription PCR (qRT-PCR) analyses (Extended Data Fig. 10c). The *BT_1675* mRNA level was around fivefold de-repressed in the absence of MasB, yet exclusively so in mid- and late exponentially growing bacteria. This suggests that the exponential phase is the critical time window when MasB exerts a negative effect on *BT_1675* and reflects the observed growth phenotype of the sRNA deletion mutant (Fig. 4b). *Trans*-complementation of MasB reverted *BT_1675* expression to near wild-type levels (Extended Data Fig. 10c). To support these findings, we harnessed a dual-plasmid fluorescence reporter assay^42,43^. The 5′ region of *BT_1675* (encompassing the predicted MasB binding site) was translationally fused to the coding sequence of superfolder green fluorescent protein (GFP) and the resulting construct transformed into *Escherichia* *coli* as a heterologous host. Co-expression of the wild-type (but not mutated; Fig. 5e, top) MasB led to a decrease of GFP intensity to ~70% compared with an unrelated control RNA (Fig. 5f and Extended Data Fig. 10d). In contrast, only the MasB variant harbouring the respective compensatory mutations was able to repress the mutated BT_1675-GFP variant in the same assay. Based on these data, we conclude that MasB inhibits the expression of the conserved tetratricopeptide domain protein BT_1675, probably through direct binding to the 5′ region of the corresponding mRNA.

### Antibiotic phenotype in light of the MasB regulatory axis

Lastly, we set out to evaluate the MasB-associated antibiotic phenotype in the context of its identified regulator (Cur) and target (BT_1675). We constructed a deletion mutant of *BT_1675* and—to test for epistatic effects—combined the single *masB* and single *cur* deletions (∆*cur*∆*masB*). Importantly, none of the mutants had a growth defect in rich medium (Extended Data Fig. 10e). When subjected to doxycycline susceptibility testing (Fig. 5g and Supplementary Table 9), ∆*cur* bacteria tended to phenocopy the enhanced antibiotic tolerance observed for the ∆*masB* mutant, whereas *trans*-complementation of Cur reverted the susceptibility to that of the isogenic wild type. Surprisingly, the ∆*cur*∆*masB* double mutant was severely affected—yet in the opposite direction to the respective single deletions—and failed to grow on doxycycline-containing plates altogether, which warrants further investigation. Deletion of the MasB target *BT_1675* had only a subtle influence on doxycycline susceptibility. Collectively, this work illustrates the synergy between transcriptomics and functional genomics for the discovery of phenotypes associated with bacterial noncoding RNAs.

## Discussion

Transcriptomics has proven invaluable for our understanding of the molecular basis of *Bacteroides*’ activities in the mammalian intestine^36,44–46^. Pinpointing the precise triggering factors that induce the expression of certain gene sets is important to understand the underlying regulatory networks and to obtain a molecular handle to rationally interfere with these processes for the benefit of the human host. However, disentangling in vivo gene expression patterns becomes complicated by the multitude of overlapping stimuli that the microbes are simultaneously exposed to in their natural habitat. To decompose host-adapted bacterial gene expression, we here reconstituted specific responses to defined environmental cues in vitro. Complementation of the corresponding transcriptomic data with functional sRNA genomics suggested specific phenotypes for 28 *B*. *thetaiotaomicron* sRNAs as starting points for targeted follow-up studies.

The predictive power of this integrative approach is exemplified by our findings on the previously uncharacterized sRNA MasB. MasB is transcriptionally activated by the master regulator Cur and, in turn, post-transcriptionally represses the mRNA for the hypothetical tetratricopeptide repeat protein BT_1675, whose function is currently unknown (Fig. 5h). A co-expression analysis approach revealed a strong positive correlation (Pearson’s *r* = 0.91) between *BT_1675* and the Gene Ontology term ‘unfolded protein binding’, comprising protein chaperones such as DnaK and GroEL (Extended Data Fig. 10f). Of interest in the present context, certain protein chaperones have the ability to stabilize resistance-conferring amino acid substitutions in drug targets^47^, suggesting that BT_1675 could play a role in the maintenance of antimicrobial resistance.

In summary, our data highlight the relevance of MasB for antibiotic sensitivity in a major human gut commensal. More generally, our study emphasizes the power of combining bacterial expression atlases with additional data modalities. Building on a state-of-the-art visualization tool, Theta-Base 2.0 allows easy and intuitive interaction with our diverse datasets and constitutes a much-needed resource for the microbiome research community.

## Methods

### Bacterial culture conditions

*Bacteroides* strains were routinely cultured in an anaerobic chamber (Coy Laboratory Products) with an anaerobic gas mix (85% N_2_, 10% CO_2_ and 5% H_2_) at 37 °C. Routine cultivation of all strains was performed in TYG medium and on Brain Heart Infusion Supplemented (BHIS) plates. For a detailed description of media composition and culture conditions for the RNA-seq analysis, refer to Supplementary Table 1.

Growth assays in the presence of diverse carbon sources were carried out in minimal medium supplemented with 0.5% of a suitable carbon source, as follows. A single colony of wild-type *B*. *thetaiotaomicron* VPI-5482 (AWS-001) was inoculated into 5 ml minimal medium–glucose and incubated anaerobically for 24 h. Then, 1 ml of this culture was centrifuged (2,000*g* for 3 min) to pellet bacterial cells that were resuspended in an equal volume of minimal medium (without a carbon source). This was subsequently used to inoculate (1:100 dilution) minimal medium containing an appropriate carbon source and incubated for the indicated time, following which aliquots (optical density equivalents of ~4) were collected for RNA extraction.

Stress response assays were performed in TYG medium as indicated below. A single colony of AWS-001 was inoculated into 5 ml TYG medium and incubated anaerobically overnight. The next day, it was sub-cultured into TYG medium and grown to the mid-exponential phase (~7 h; OD_600_ = 2.0). This culture was sub-divided into 5 ml fractions corresponding to each stress condition and centrifuged to pellet the bacterial cells, as before. The pellet was resuspended in an equal volume of TYG medium containing the indicated concentration of a stressor and incubated for a further 2 h, following which samples were collected for RNA extraction.

### Bacterial genetics

A detailed list of the strains, plasmids and oligonucleotides used in this study can be found in Supplementary Table 10. To create ∆*masB*, we employed a previously established method^48^. To this end, we assembled 1-kilobase sequences flanking the deletion site into the pExchange-*tdk* suicide vector and introduced this construct into *E*. *coli* S17-1 λpir. The resulting transformants were mated with *B. thetaiotaomicron* Δ*tdk* (AWS-003) and the resulting conjugants were selected on 5-fluoro-2′-deoxyuridine plates. Single recombinants were isolated on BHIS agar with 200 μg ml^−1^ gentamicin and 25 μg ml^−1^ erythromycin (BHIS^gent/erm^). Double recombinants, leading to either scarless deletion mutants or wild-type revertants, were identified by their ability to grow on BHIS agar with 200 μg ml^−1^ 5-fluoro-2′-deoxyuridine while being unable to grow on BHIS agar with 25 μg ml^−1^ erythromycin. The *masB* complementation strain (*masB*^+^) was assembled using a version of the pNBU2 vector system, as previously described^48^. The complete *masB* gene sequence was integrated into pNBU2 using Gibson Assembly (New England Biolabs (NEB)) to ensure transcription from the native TSS. This construct was conjugated into the ∆*masB* strain via *E*. *coli* S17-1 λpir, as described above.

The other deletion mutants (∆*cur*, ∆*1675* and ∆*cur*∆*masB*) were generated using the pSIE1 plasmid system^49^. Briefly, 750-nucleotide flanking regions around the deletion site were Gibson assembled (NEB) into the linearized pSIE1 plasmid (*Spe*I and *Bam*HI digested). The assembled plasmid was subsequently introduced into *B*. *thetaiotaomicron* via conjugation with *E*. *coli* S17-1 λpir and the conjugants were streaked onto BHIS^gent/erm^ plates. Resistant colonies were cultured overnight in TYG medium without antibiotics, and dilutions (10^−1^ to −3) were plated onto BHIS agar with 100 ng ml^−1^ anhydrotetracycline (aTC). Colony PCR and Sanger sequencing were used to confirm the intended deletions. The *cur*^+^ complementation strain resulted from Gibson Assembly (NEB) of full-length *cur* with pWW3452 (AWP-015) such that transcription was under the control of the phage promoter on the plasmid. It was ensured that the 3′ end of the transcript maintained a reading frame with the downstream FLAG and His tags. The construct was conjugated into the ∆*cur* background as described above.

### Total RNA purification and removal of genomic DNA

All of the RNA-seq samples were collected as biological duplicates. Total RNA was isolated by the hot-phenol method, as follows. Briefly, bacterial cultures containing a total of ~4 OD equivalents of cells were collected and a one-fifth volume of stop mix was added (5% vol vol^−1^ water-saturated phenol; pH > 7.0; 95% vol vol^−1^ ethanol)^50^. Cell lysis was achieved by incubation with lysozyme (600 µl; 0.5 mg ml^−1^) and sodium dodecyl sulfate (60 µl of a 10% solution) for 2 min at 64 °C with the subsequent addition of NaOAc (66 µl of a 3 M solution). Extraction with 750 μl phenol (ROTIAqua-Phenol) was carried out at 64 °C for 6 min, followed by the addition of 750 μl chloroform. Precipitation of RNA from the aqueous phase was achieved with twice the volume of ethanol and 3 M NaOAc (30:1) mix and incubated at −80 °C overnight. The samples were then centrifuged and the pellets washed with ethanol (75% vol vol^−1^), followed by resuspension in 50 µl RNase-free water. Traces of genomic DNA were removed by treating ~40 µg total RNA with 5 U DNase I (Fermentas) and 0.5 µl SUPERase·In RNase Inhibitor (Ambion) in a reaction volume of 50 µl. Samples for dRNA-seq were prepared by pooling equimolar amounts (each 100 ng) of total RNA from each condition.

### cDNA library preparation and sequencing

For dRNA-seq, samples were treated according to a previous protocol^16^. Before synthesizing cDNA, pooled total RNA was fragmented by ultrasound (four pulses of 30 s each at 4 °C) and then treated with T4 Polynucleotide Kinase (NEB). The RNA sample was then split evenly, one half of which was treated with Terminator Exonuclease to enrich for primary transcripts, whereas the other half remained untreated. The samples were then poly(A)-tailed using poly(A) polymerase and the 5′-PPP removed using 5′ polyphosphatase (Epicentre Biotechnologies). RNA adaptors were ligated and the synthesis of first-strand cDNA was performed using M-MLV reverse-transcriptase and oligo(dT) primers. The cDNA was subsequently amplified to a concentration of ~10–20 ng µl^−1^, purified (Agencourt AMPure XP kit; Beckman Coulter Genomics) and fractionated in a size range of 200–600 bp. The libraries were deep-sequenced on an Illumina NextSeq 500 system using 75 bp read length at Vertis Biotechnologie.

For conventional RNA-seq, samples were first depleted of ribosomal RNA (rRNA) using the Pan-Prokaryote riboPOOL kit (siTOOLs Biotech). This involved incubation of 1 µg total RNA with 100 pmol rRNA-specific biotinylated DNA probes at 68 °C for 10 min, followed by a shift to 37 °C for 30 min in 0.25 mM ethylenediaminetetraacetic acid (EDTA), 2.5 mM Tris-HCl (pH 7.5) and 500 mM NaCl. Depletion of rRNA–DNA hybrids was achieved by two 15-min incubation periods with streptavidin-coated magnetic Dynabeads MyOne Streptavidin C1 beads (0.45 mg; Thermo Fisher Scientific) in 0.25 mM EDTA, 2.5 mM Tris-HCl (pH 7.5) and 1 M NaCl at 37 °C. The samples were then purified using the Zymo RNA Clean & Concentrator kit along with DNase I treatment (Zymo Research). Libraries were prepared with the NEBNext Multiplex Small RNA Library Prep kit for Illumina (NEB) according to the manufacturer’s instructions and the following modifications. Samples were fragmented at 94 °C for 2.75 min, per the NEBNext Magnesium RNA Fragmentation Module (NEB) with subsequent RNA purification using the Zymo RNA Clean & Concentrator kit. The fragmented RNA was then 3′ dephosphorylated, 5′ phosphorylated and decapped with 10 U T4 Polynucleotide Kinase ± 40 nmol ATP and 5 U RNA 5′ Pyrophosphohydrolase (NEB). After each step, RNA was purified as mentioned above. The fragmented RNA was then ligated to adapters (3′ SR and 5′ SR, pre-diluted 1:3 in nuclease-free water) and the cDNA was amplified for 14 cycles. These barcoded libraries were purified using MagSi-NGSPREP Plus beads (AMSBIO) at a 1.8:1 ratio of beads to sample volume. Libraries were checked for quantity and quality using a Qubit 3.0 Fluometer (Thermo Fisher Scientific) and a 2100 Bioanalyzer with the High Sensitivity DNA kit (Agilent). Pooled libraries were sequenced on the NextSeq 500 platform (Illumina) at the Core Unit SysMed of the University of Würzburg.

### Read processing and mapping

Generated reads were quality checked using FastQC (version 0.11.8) and adapters were trimmed using Cutadapt (version 1.16) with Python (version 3.6.6), using the following parameters: -j 6 -a Illumina Read 1 adapter=AAGATCGGAAGAGCACACGTCTGAACTCCAGTCA -a Poly A=AAAAAAAAAAA --output=out1.fq.gz --error-rate=0.1 --times=1 --overlap=3 --minimum-length=20 --nextseq-trim=20 3_1. For both sequencing data types (dRNA-seq and conventional RNA-seq), READemption^51^ (version 0.4.5) was used to map reads to the *B*. *thetaiotaomicron* VPI-5482 reference genome (NC_004663.1) and plasmid (NC_004703.1). Details of the alignment statistics can be found in Supplementary Table 11.

### Transcriptome annotation

We used the ANNOgesic pipeline^27^ (version 0.7.33) to update the annotations of TSSs, terminators, operons and noncoding RNAs, as previously described^16^. In short, TSSs were identified using the TSSpredator function, which compares the relative enrichment of reads between Terminator Exonuclease-treated and untreated libraries to identify enriched peaks that are characteristic of the protected 5′ ends of primary transcripts. TSSs were classified on the basis of this enrichment and distance, relative to a coding gene. Primary TSSs were identified as having the highest enrichment within a 300-bp region upstream of an open reading frame. All other TSSs within this region were classified as secondary TSSs. Internal TSSs were defined as originating on the sense strand within a coding sequence, whereas antisense TSSs were those that originated on the antisense strand and overlapped with or within 100-bp flanks of a sense gene. All remaining TSSs were classified as orphan TSSs. To predict terminators, ANNOgesic utilizes two heuristic algorithms, one of which scans the genome for Rho-independent terminators (TransTermHP) and the other predicts terminators by detecting a decrease in read coverage between two adjacent genes. Leveraging the wealth of our diverse conditional datasets, we additionally utilized the operon detection function of ANNOgesic (default settings) to predict both operons and sub-operons based on the other detected features; namely, TSSs, transcripts and genes supplied as general feature format (GFF) files.

The automated prediction of noncoding RNAs was done using the srna function of ANNOgesic, which first compares predicted transcripts with known RNAs within the sRNA database (all sequences were downloaded from BSRD^52^) and the non-redundant protein database (ftp://ftp.ncbi.nih.gov/blast/db/FASTA/). Candidates that were contained in the sRNA database were retained, while those contained in the non-redundant protein database were excluded from further analysis. All remaining transcripts were classified as intergenic sRNA candidates if they possessed a defined TSS, stable secondary structure (RNAfold normalized folding energy < −0.05) and length within 30–500 nucleotides and did not overlap with any other genetic feature in either sense or antisense orientation. The prediction of *cis*-asRNAs was based on the same criteria along with the presence of an annotated gene on the opposing strand. Untranslated region (UTR)-derived sRNAs were classified as 5′ if they shared the TSS with an mRNA and were associated with a read coverage drop or processing site in front of the coding sequence. Similarly, 3′ UTR-derived sRNAs were predicted by either a TSS or processing site in the 3′ UTR and either a processing site or terminator shared with an mRNA. Intra-operonic sRNAs were associated with a TSS or processing site at the 5′ end and a read coverage drop or processing site at the 3′ end.

As with all computationally automated predictions, manual curation was necessary to ensure the accuracy of the global annotations. Manual confirmation of sRNA predictions was guided by the following criteria for excluding putative sRNA annotations: (1) lack of an identifiable promoter or processing site up to 50 bp upstream of a predicted sRNA’s 5′ end; (2) complete overlap with an annotated mRNA in the most recent genome update on the National Center for Biotechnology Information’s Nucleotide database (NC_004663.1; 21 August 2022); (3) complete overlap with an annotated terminator sequence; (4) overlap with *cis*-regulatory elements such as riboswitches or RNA thermometers; and (5) no evident change in read coverage relative to flanking regions. As a result, we report a total of 135 intergenic sRNAs in *B*. *thetaiotaomicron*. This includes an overlap of 91 sRNAs previously identified^16^, as well as 44 novel sRNA candidates. In total, 20 candidates previously annotated as intergenic sRNAs^16^ were reassigned here to different sub-classes (Supplementary Table 12): 11 were re-annotated as asRNAs (as a divergently encoded genetic feature was discovered in the present data), three were re-annotated as 3′ UTR-derived sRNAs (due to their overlap with the 3′ end of an mRNA) and six were re-annotated as 5′ UTR-derived sRNAs (as the present dataset allowed us to refine TSS annotations). Additionally, 14 previously annotated sRNA candidates^16^ were eliminated as they were very weakly expressed in the former dataset and their existence was not further supported by the present dataset, despite a greater sequencing depth.

Updated annotations of the TSSs, terminators, operons and noncoding RNAs can be accessed via Theta-Base 2.0 (http://micromix.helmholtz-hiri.de/bacteroides/). Coding genes and sRNAs can be interrogated using either their ID (BT_xxxx or BTncxxx) or—when applicable—their trivial name (for example, MasB).

### Prediction of invertible DNA regions

Invertible DNA regions were predicted using the PhaseFinder -locate pipeline as described in ref. ^28^. Briefly, inverted repeats were determined by allowing no mismatches for repeats of a maximum of 11 bp, one mismatch for repeats up to 13 bp and two mismatches for repeats with lengths exceeding 19 bp. Homopolymeric inverted repeats were removed and the maximum GC content per inverted repeat was filtered to be between 15 and 85%.

### Differential gene expression analysis

Differential gene expression analysis was performed using the *R* package edgeR (version 3.38.2)^53,54^. Using the filterByExpr function, the genes with a counts per million (CPM) value of >0.6635 (equivalent to around ten reads per sample) across all replicates under each growth condition (median library size of ~15 million reads) were retained for differential analysis. While calling for contrasts (using the makeContrast function), the analysis was sub-divided into two groups based on the respective control condition: all conditions with varying carbon sources (including starvation) were compared with glucose as a control, whereas all stress conditions were compared with the condition immediately before stress induction (that is, a mid-exponential phase culture in TYG medium). Differential gene expression data across all conditions relative to their respective control condition can be found in Supplementary Table 4.

### Gene set annotation and enrichment analyses

We assembled a list of functionally annotated gene sets from the literature. We recovered annotations of PULs 01–96 from the Polysaccharide Utilization Loci Database^4^; CPSs and conjugative transposons from ref. ^55^; the genes transcribed from promoter motifs PM1 and PM2 from ref. ^16^; regulons from RegPrecise version 3.2 (ref. ^56^); the Cur regulon from ref. ^13^; annotated KEGG pathways and modules from the KEGG database (accessed on 1 December 2022); Gene Ontology terms from UniProt (accessed on 25 November 2021); and predicted KEGG modules and pathways and Gene Ontology terms from an eggNOG version 5.0 (ref. ^57^) annotation of the *B*. *thetaiotaomicron* genome. Gene set enrichment analysis was performed with the fgsea *R* package over all gene sets with more than nine genes, except for PULs, which were retained irrespective of their gene number. Genes were ranked based on the −log_10_[*P* value] × sign[FC] metric.

### Northern blot

Northern blotting was performed as described previously^16^. In short, total RNA (2.5–10 µg) was electrophoretically resolved on a 6% (vol vol^−1^) polyacrylamide (PAA) gel containing 7 M urea and electro-blotted onto a membrane (Amersham Hybond-XL) at 50 V and 4 °C for 1 h. The blots were probed with gene-specific ^32^P-labelled oligonucleotides in Hybri-Quick buffer (Carl Roth) at 42 °C and subsequently exposed to a phosphor screen as required. Images were visualized using a phosphorimager (FLA-3000 Series; Fuji).

### Reanalysis of TIS data

We reanalysed fitness data from a comprehensive *B*. *thetaiotaomicron* transposon mutant library that probed a suite of hundreds of different conditions, including 48 different carbon sources and 56 stress-inducing compounds^19^, in the context of our extensive noncoding RNA annotation (see above). This was done with the primary objective of identifying and possibly correlating gene expression from our transcriptomic dataset with mutant fitness data and thereby allowing us to draw biologically meaningful conclusions. To further streamline our analysis, we focused exclusively on independently encoded intergenic sRNAs since phenotypes pertaining to such mutants would probably not involve polar effects. Consequently, of the 135 intergenic sRNAs identified in *B*. *thetaiotaomicron*, we obtained fitness data for 81 sRNAs (~70%), of which 28 were associated with statistically significant effects (|*t*| > 4) in at least one successful experiment (Supplementary Table 8). A successful experiment requires that each gene is represented by a sufficient number of barcode counts^58^. The fitness of a gene is defined as the average log_2_[change in relative abundance of its mutants (|fit|)]. Negative and positive values mean that the sRNA mutants were less or more fit, respectively, than the average strain in the pool. Experiments with ‘jackpot’ effects, whereby the disruption of an sRNA resulted in a large competitive advantage versus the other mutants in the pool, were retained, but specifically labelled as strong phenotypes (|fit| > 2 and |*t*| > 5) (Supplementary Table 8). A third category, namely ‘combined’, comprised those phenotypes that were both strong and significant, per the above criteria.

### Launch of Theta-Base 2.0

Theta-Base 2.0 (http://micromix.helmholtz-hiri.de/bacteroides/) was created using Micromix (https://github.com/BarquistLab/Micromix)^59^, which relies on Flask^60^ (back end) and Vue.js (front end), storing underlying visualization and expression data using MongoDB. The Clustergrammer plugin uses the API from the Ma’ayan laboratory^61^, while the heat map plugin follows the same front- and back-end architecture as the main site. Gene set annotations (Gene Ontology terms, KEGG pathways and modules, PULs, CPSs, conjugative transposons, promoter motifs and known regulons) were prepared as described in the section ‘Gene set annotation and enrichment analyses’ and can be found in Supplementary Table 3. The sRNA fitness dataset was adapted from Supplementary Table 8. Deployment of the back and front ends uses Gunicorn (https://readthedocs.org/projects/gunicorn-docs) and Nginx^62^.

*B*. *thetaiotaomicron* datasets can be manually selected by first clicking the Bacteroides Theta tab, followed by providing a title and then selecting an appropriate dataset using the dropdown menu. Users can select from a choice of expression data (that is, normalized in CPM or log_2_[FC] (compared with control conditions)) or between the entire dataset or specific sRNA fitness data. As an option, columns of interest can be further customized using the ‘Select columns’ box and by subsequently clicking the ‘Add’ tab. Users also have the option to add their own data, as outlined in additional tabs, such as by uploading a delimited file.

The resulting data tables are displayed in the browser and can be filtered or transformed. For example, the ‘Filter’ button allows data tables to be filtered using keywords with prompts to make the search process seamless. The ‘Functional annotation’ button permits the user to select from a large number of preset manually curated gene lists, such as ‘GO term’, ‘KEGG pathway’, ‘PUL’, ‘CPS’ and ‘CTn’, to name a few. A third button, ‘ncRNA’ permits selection of manually curated noncoding RNA gene sets (for example, ‘High-confidence intergenic sRNAs’, ‘Intergenic sRNAs’ and ‘*Cis*-antisense RNAs’). Once the desired genes have been filtered, they may be transformed by clicking the ‘Transform data’ button and performing operations such as rounding values, log conversion or calculating transcripts per million. Once datasets have been loaded by the user, they can be further examined using three visualization modes; namely, ‘Heatmap’, ‘Clustergrammer’ and ‘JBrowse’. Two- and three-dimensional heat maps can be generated using the Heatmap function. Note that the heat map defaults to a three-dimensional option, but users can manually switch to the two-dimensional option. Heat maps can be customized using the menu on the left that permits changes to the colour gradients and overall structure. Customized heat maps can be downloaded in SVG or PNG formats using the download tab. Alternatively, for clustering according to genes or conditions, ‘Clustergrammer’ is recommended. Selecting this tab generates a two-dimensional dynamic heat map of the data that can be further investigated using the menu on the left. Currently, the tool only permits a maximum of 200 rows to be loaded and users will be notified if more rows are selected. Customized heat maps and data tables can be downloaded using the ‘Take snapshot’ and ‘Download matrix’ buttons, respectively. For a detailed view of normalized coverage plots for the investigated conditions, in addition to those published in the first iteration of Theta-Base^16^, users can select the ‘JBrowse’ button^63^. Users are free to select from a range of updated annotations displaying high-resolution maps for noncoding RNAs (ncRNA), TSSs (TSSv3), terminators (term_v2), operons (Operon_structure), a transposon insertion map related to the fitness data (Tn_insertions) and invertons (Inverted_repeats).

On the top right of the website there are four buttons. The ‘Padlock’ button locks the current state of the site, allowing users to copy their URL and share with colleagues. The next (‘Download’) button allows users to download the currently selected dataset as an Excel or a delimited file (such as .csv). The ‘New document’ button will re-load the website so users can select another dataset. The ‘Help’ button—when clicked—will provide pop-over text explaining various features of the site.

### Antibiotics growth curve analyses and agar strip assays

Bacterial growth curves were determined by inoculating a single colony each of AWS-003 (Δ*tdk*; referred to as the wild-type in Fig. 4) and AWS-029 (∆*masB*) into 5 ml TYG medium and incubating overnight under anaerobic conditions. These cultures were sub-cultured (1:100 dilution) in 2 ml TYG medium containing the indicated final concentrations of doxycycline (Sigma-Aldrich), tetracycline (AppliChem) or a water control. The samples (200 µl volume) were incubated in a 96-well flat-bottom plate (Nunclon) at 37 °C (doxycycline) or 40 °C (tetracycline) with continuous shaking (double orbital) in a microplate spectrophotometer (BioTek Epoch 2). Optical densities were recorded every 20 min. The assay was performed in three biological replicates, each comprising technical duplicates.

Antibiotics strip assays were performed by dipping a sterile cotton swab into overnight TYG cultures of AWS-003 or AWS-029 and streaking on BHIS agar plates containing strips for doxycycline (EM103 (HiMedia; in Fig. 4c) or 92156 (Liofilchem; in Fig. 5g)) or tetracycline (EM056; HiMedia). The plates were incubated anaerobically for 48 h at 37 °C and images were taken. The minimal inhibitory concentrations were derived from the positions where the inhibition ellipses intersected the strips.

### Gene co-expression analyses

Correlation of the expression of all *B*. *thetaiotaomicron* genes across all of the profiled carbon source and stress conditions was calculated by generating a correlation matrix (Pearson’s correlation score) of the *z* scores of the CPM values of each gene. To identify the correlation in expression between our gene sets (see ‘Gene set annotation and enrichment analyses’) and a given gene of interest (MasB in Fig. 5a and *BT_1675* in Extended Data Fig. 10f), the median of the correlation values between all genes within a gene set and the gene of interest was calculated. Gene sets composed of fewer than ten operons were excluded from this analysis.

### MS2 affinity purification and sequencing

A *B*. *thetaiotaomicron* Δ*masB* mutant complemented with either MS2-MasB (AWS-062) or untagged MasB (AWS-036) was diluted 1:100 in TYG medium from an overnight culture grown anaerobically at 37 °C. At an OD_600_ of 2.0, expression of MS2-tagged MasB and untagged MasB was induced by the addition of 200 ng ml^−1^ aTC. After another 2 h of growth at 37 °C, 90 OD equivalents of the cultures were collected, centrifuged for 20 min at 2,000*g* and 4 °C and snap-frozen in liquid nitrogen. MS2 pulldown and RNA purification was performed as described in ref. ^64^, but with slight modifications to adapt the protocol to *Bacteroides*. Specifically, the column was washed only six (instead of eight) times with buffer A before elution. Elution itself was then induced with 600 µl (rather than 300 µl) elution buffer.

For library preparation (at Vertis Biotechnologie), the RNA samples were first fragmented using ultrasound (four pulses of 30 s each at 4 °C). Then, an oligonucleotide adapter was ligated to the 3′ ends of the RNA molecules. First-strand cDNA synthesis was performed using M-MLV reverse-transcriptase and the 3′ adapter as a primer. The first-strand cDNA was purified and the 5′ Illumina TruSeq sequencing adapter was ligated to the 3′ end of the antisense cDNA. The resulting cDNA was PCR-amplified to ~10–20 ng μl^−1^ using a high-fidelity DNA polymerase. The cDNA was purified using the Agencourt AMPure XP kit (Beckman Coulter Genomics) and analysed by capillary electrophoresis. For Illumina sequencing, the samples were pooled in approximately equimolar amounts. To deplete sequences derived from 5S rRNA, the cDNA pool was digested using probes specific for bacterial 5S and Cas9 endonuclease. Afterwards, the cDNA pool was fractionated in the size range of 200–600 bp using a preparative agarose gel. An aliquot of the size-fractionated pool was analysed by capillary electrophoresis. The cDNA pool was sequenced on an Illumina NextSeq 500 system using a read length of 2 × 150 bp.

Generated reads were quality-checked using FastQC (version 0.11.8) and adapters were trimmed using BBDuk with the following parameters: qtrim=r trimq=10 ktrim=r ref=bbmap/ressources/adapters.fa k=23 mink=11 hdist=1 tpe tbo. BBmap was used to map reads to the *B*. *thetaiotaomicron* VPI-5482 reference genome (NC_004663.1) and plasmid (NC_004703.1), as well as to the MS2-MasB sequence. Read quantification was performed using featureCounts (2.0.1). Differential abundance analysis between the MS2-MasB and untagged samples was conducted using edgeR^65^ in combination with RUVSeq^66^ to estimate the factor of unwanted variation using replicate sample with correction factor k=1.

### IntaRNA prediction of sRNA–mRNA interactions

In silico interaction prediction between MasB and its putative mRNA targets *fusA2* and *BT_1675* was performed with the help of IntaRNA^40,41^ using the Vienna RNA package (2.4.14 and boost 1.7) at default settings along with the output flag (--out=pMinE:FILE.csv) to generate minimal energy values for intermolecular index pairs. For visualization, the resulting values were plotted in form of a heat map in *R* (version 4.2).

### In vitro transcription and radiolabelling of RNA

DNA templates for in vitro transcription were amplified using genomic DNA and primer pairs carrying a T7 promoter (Supplementary Table 10). The in vitro transcription reaction was performed using the MEGAscript T7 kit (Thermo Fisher Scientific) followed by DNase I digestion (1 U; 37 °C; 15 min). RNA products were then excised from a 6% (vol vol^−1^) PAA-7M urea gel by comparison with a Low Range RNA ladder (Thermo Fisher Scientific) and eluted overnight in elution buffer (0.1 M NaOAc, 0.1% sodium dodecyl sulfate and 10 mM EDTA) on a thermoblock at 8 °C and 1,400 r.p.m. The next day, the RNA was precipitated in an ethanol:NaOAc (30:1) mix, washed with 75% ethanol and resuspended in 20 µl water (at 65 °C for 5 min).

Radioactive labelling of the in vitro-transcribed RNA was carried out by dephosphorylating 50 pmol RNA with 25 U calf intestine alkaline phosphatase (NEB) in a 50 µl reaction and incubating at 37 °C for 1 h. The dephosphorylated RNA was extracted using phenol:cholorform:isoamylalcohol (25:24:1) and precipitated as described above. Next, 20 pmol of this RNA was 5′ end-labelled (20 µCi ^32^P-γATP) using 1 U polynucleotide kinase (NEB) at 37 °C for 1 h in a 20 µl reaction. The labelled RNA was purified using a G-50 column (GE Healthcare) and extracted from a PAA gel as described above.

### EMSA

EMSA was carried out in a reaction volume of 10 μl, containing 1× RNA structure buffer (Ambion), 1 μg yeast RNA (~4 μM final concentration), 5′ end-labelled MasB RNA (4 nM final concentration) and an mRNA segment of 137 nucleotides in length, spanning the predicted MasB target site within *BT_1675* (see Fig. 5e) at final concentrations of 0, 8, 16, 32, 64, 128, 256, 512 and 1,024 nM. Following incubation at 37 °C for 1 h, 3 μl of 5× native loading dye (0.2% bromophenol blue, 0.5× TBE and 50% glycerol) was added to each tube. All of the samples were loaded on a native 6% (vol vol^−1^) PAA gel in 0.5× TBE buffer and run at 300 V and 4 °C for 3 h. The gel was dried, exposed and visualized using a phosphorimager (FLA‐3000 Series; Fuji). The experiment was repeated three times and quantified using ImageJ version 1.52s^67^ and GraphPad Prism version 9 for Windows (GraphPad Software; www.graphpad.com). The dissociation constant (*K*_d_) was calculated via the one site-specific binding formula:

$$Y=B_{{\rm{max}}} \times X/({K_{{\rm{d}}}}+X)$$

where *Y* is the specific binding; *X* the concentration of radio ligand; *B*_max_ the maximum binding in the same unit as *Y*; and *K*_d_ the dissociation constant in the same unit as *X*.

### qRT-PCR analysis

For the qRT-PCR assays, Δ*tdk B*. *thetaiotaomicron* (AWS-003; referred to as the wild-type in Fig. 5), Δ*masB* (AWS-029) and *masB*^+^ (AWS-036) were grown anaerobically overnight at 37 °C in 5 ml TYG medium, then sub-cultured 1:100 in TYG and induced with 200 ng ml^−1^ aTC. Around four optical density equivalents of samples were collected at the early exponential phase (OD_600_ = 0.3), mid-exponential phase (OD_600_ = 2.0) and late exponential phase (OD_600_ = 3.7) for RNA extraction, as described above. For the starvation condition, the same strains were grown anaerobically for 24 h at 37 °C in 5 ml minimal medium supplemented with 0.5% glucose, and then sub-cultured 1:100 in 0.5% glucose-containing minimal medium supplemented with 200 ng ml^−1^ aTC. At OD_600_ = 2.0, the cultures were centrifuged, the supernatant was discarded and the pellet was resuspended in minimal media without a carbon source and incubated anaerobically at 37 °C for another 2 h. Around four optical density equivalents of the samples were collected for RNA extraction. qRT-PCR reactions were performed as described in ref. ^16^. A minimum of three biological replicates were pipetted and plates were analysed on a QuantStudio 5 instrument (Thermo Fisher Scientific).

### Dual-plasmid fluorescence reporter assay

Strains of *E*. *coli* TOP10, which were engineered to carry translational fusions of superfolder GFP^43^ to different variants of the 5′ part of the *BT_1675* coding sequence, were cultured in Lysogeny Broth medium supplemented with chloramphenicol (20 μg ml^−1^) and carbenicillin (100 μg ml^−1^) until an OD_600_ of 0.5 was reached. Subsequently, 100 μl of the cultures was collected and subjected to three washes with 1× phosphate-buffered saline before fixation with a 4% paraformaldehyde solution. The fluorescence intensity of GFP was measured in phosphate-buffered saline using flow cytometry (NovoCyte Quanteon; Agilent).

### Statistics and reproducibility

Conventional RNA-seq of diverse growth conditions, dRNA-seq of pooled conditions and MAPS were performed in biological duplicates. Testing for differential expression or enrichment was performed using the generalized linear model likelihood ratio test implemented in edgeR^53^. Northern blots were performed in two biological replicates. EMSAs were performed in technical triplicates. qRT-PCR analysis was performed in a minimum of three biological replicates, each comprising technical duplicates, and a Mann–Whitney test was used to call significant comparisons. Growth curve experiments were performed in a minimum of three biological replicates, unless explicitly mentioned otherwise. Two-plasmid GFP reporter assays were performed in biological triplicates and Tukey’s multiple comparisons test was used to test for statistically significant differences. Minimal inhibitory concentration strip assays were performed in a minimum of five biological replicates for doxycycline and three biological replicates for tetracycline. No statistical method was used to predetermine the sample size. Instead, sample sizes were chosen based on previous experience and studies^16^. No data were excluded from the analyses. The experiments were not randomized. The investigators were not blinded to allocation during the experiments and outcome assessment.

### Reporting summary

Further information on research design is available in the Nature Portfolio Reporting Summary linked to this article.

## Supplementary information

Supplementary Information Supplementary text.

Reporting Summary

Supplementary Tables Supplementary Tables 1–12.

## Source data

Source Data Fig. 2 Expression profiling data for Fig. 2a–c,e,f.

Source Data Fig. 3 Expression data for Fig. 3b.

Source Data Fig. 3 Unmodified gels for Fig. 3b.

Source Data Fig. 4 Growth curve data for Fig. 4b.

Source Data Fig. 5 Source data for Fig. 5a,d–g.

Source Data Fig. 5 Unmodified blots for Fig. 5b,e.

Source Data Extended Data Fig. 1 Growth curve data for Extended Data Fig. 1a–f.

Source Data Extended Data Fig. 5 Expression profiling data for Extended Data Fig. 5a–m.

Source Data Extended Data Fig. 6 Expression profiling data for Extended Data Fig. 6a,c,d.

Source Data Extended Data Fig. 7 Expression profiling and statistical data for Extended Data Fig. 7a,c.

Source Data Extended Data Fig. 7 Unmodified blots for Extended Data Fig. 7b.

Source Data Extended Data Fig. 8 Growth curve data for Extended Data Fig. 8b,c.

Source Data Extended Data Fig. 8 Unmodified blots for Extended Data Fig. 8d.

Source Data Extended Data Fig. 9 Growth curve data for Extended Data Fig. 9c.

Source Data Extended Data Fig. 9 Unmodified blots for Extended Data Fig. 9d,e.

Source Data Extended Data Fig. 10 qRT-PCR data for Extended Data Fig. 10c, growth curve data for Extended Data Fig. 10d and gene set enrichment data for Extended Data Fig. 10e.

Source Data Extended Data Fig. 10 Unmodified blots for Extended Data Fig. 10c.

## Data Availability

The raw sequencing data are available from the National Center for Biotechnology Information’s Gene Expression Omnibus (http://www.ncbi.nlm.nih.gov/geo) under the accession number GSE234958. Our analysed sequencing data are accessible at http://micromix.helmholtz-hiri.de/bacteroides/. Source data are provided with this paper.

## Code availability

Core software central to the conclusions drawn in this study is publicly available and its usage parameters described in the appropriate sections above. Code for the Micromix data integration platform is available at https://github.com/BarquistLab/Micromix (ref. ^59^).
