# Supplementary material for: An expanded transcriptome atlas for Bacteroides thetaiotaomicron reveals a small RNA that modulates tetracycline sensitivity
Source: Nat Microbiol. 2024 Mar 25;9(4):1130–44. doi: 10.1038/s41564-024-01642-9 (PMC10994844; doi:10.1038/s41564-024-01642-9)
Supplement: Supplementary file 1 — Supplementary text. [file 41564_2024_1642_MOESM1_ESM.pdf]

nature microbiology

Resource

https://doi.org/10.1038/s41564-024-01642-9

An expanded transcriptome atlas for *Bacteroides thetaiotaomicron* reveals a small RNA that modulates tetracycline sensitivity

In the format provided by the
authors and unedited

SUPPLEMENTARY TEXT

Stress-specific RNA-seq data confirm previous results from other labs

Stress-specific "marker genes" inferred from the literature showed the anticipated alterations [75,76], including the induction of *BT_1606* (encoding cytochrome C peroxidase) and *BT_1456* (thioredoxin) when bacterial cultures were shifted to an aerobic environment (Extended Data Figure 5d). When exposed to deoxycholate or the bile salt cocktail, the most highly expressed operons included *BT_2792–BT_2795* and *BT_0691–BT_0692* (Fig. 2c; Extended Data Figure 5e, g) that are both known to contribute to *Bacteroides* fitness under bile stress [19,70]. In contrast, the genes for the bile salt hydrolases BSHa (*BT_1259*) and BSHb (*BT_2086*), which decrease *Bacteroides* survival in the presence of conjugated bile acids [48], were downregulated under those conditions (Fig. 2c; Extended Data Figure 5e).

Carbon source-specific RNA-seq data are supported by the existing literature

Maltose serves as the inner membrane signal that is recognized by SusR, which subsequently upregulates the genes of the starch utilization system (Sus = PUL66) [71,72,77]. In our data, maltose consumption was reflected by the specific upregulation of *sus* genes (avg. log2FC relative to glucose = 7.1; Extended Data Figure 5j). In the presence of xylose, the corresponding utilization operon (*BT_0791-BT_0794*) [13] was induced (avg. log2FC = 6.8; Extended Data Figure 5i) along with PUL37 (avg. log2FC = 4.5) and, to lesser extent, PUL80 (avg. log2FC = 3.9). Arabinose specifically triggered expression of arabinose utilization operons [12] (Extended Data Figure 5h) and of PUL07 (avg. log2FC compared to glucose = 5.1) (Fig. 2b), which was previously found upregulated in the presence of arabinan or pectic galactan [34]. Functional analysis additionally identified genes belonging to the regulon governed by AraR (BT_0354)—the transcriptional regulator of L-arabinose catabolism [78]—to be enriched among the differentially expressed genes in arabinose-containing medium (Extended Data Figure 3b; Supplementary Table 5). During growth on GlcNAc, which forms a part of the mucin structure [79], PUL80—previously shown to be induced in mucin [7,11]—was induced (avg. log2FC = 4.5) (Extended Data Figure 5k). A gene set enrichment analysis additionally identified PUL20 that is involved in the processing of complex mammalian glycans [7]. S1 sulfatase genes, required to process the heavily sulfated intestinal glycoproteins [50], belonged to the most highly mucin-induced genes (Fig. 2e). Bacteria deprived of nutrients activated the stringent response to reallocate resources away from growth and favor persistence (Extended Data Figure 3b; downregulation of gene sets related to translation, peptidoglycan biosynthesis, and cell cycle). Starved bacteria further induced expression of the arabinose utilization gene *araM* (*BT_0356*) and its associated arabinose-utilizing PUL07 (avg. log2FC = 3.1; Extended Data Figure 5l), which is all in line with previous reports [12,73] (Extended Data Figure 5m).

Establishment of MAPS in B. thetaiotaomicron

We fused a tandem MS2 aptamer to the 5' end of MasB, as *in silico* RNA folding suggested this would maintain the native sRNA secondary structure (Extended Data Figure 9a). The strain expressing the tagged version of the sRNA from an inducible promoter in the Δ*masB* background did not show a growth defect as compared with wild-type bacteria (Extended Data Figure 9b, c). A strain expressing the untagged sRNA was included as a control (Extended Data Figure 9d). After optimizing the affinity purification protocol for *Bacteroides* (see Methods; Extended Data Figure 9e), we subjected the co-purified RNA samples from two independent pulldown experiments to cDNA library preparation and sequencing (Extended Data Figure 9f).
