# Supplementary material for: An expanded transcriptome atlas for Bacteroides thetaiotaomicron reveals a small RNA that modulates tetracycline sensitivity
Source: Nat Microbiol. 2024 Mar 25;9(4):1130–44. doi: 10.1038/s41564-024-01642-9 (PMC10994844; doi:10.1038/s41564-024-01642-9)
Supplement: Supplementary file 2 — Reporting Summary [file 41564_2024_1642_MOESM2_ESM.pdf]

nature portfolio

Corresponding author(s): Alexander J. Westermann

Last updated by author(s): Jan 29, 2024

Reporting Summary

Nature Portfolio wishes to improve the reproducibility of the work that we publish. This form provides structure for consistency and transparency in reporting. For further information on Nature Portfolio policies, see our Editorial Policies and the Editorial Policy Checklist.

Statistics

For all statistical analyses, confirm that the following items are present in the figure legend, table legend, main text, or Methods section.

n/a	Confirmed	
☐	☒	The exact sample size (*n*) for each experimental group/condition, given as a discrete number and unit of measurement
☐	☒	A statement on whether measurements were taken from distinct samples or whether the same sample was measured repeatedly
☐	☒	The statistical test(s) used AND whether they are one- or two-sided<br>*Only common tests should be described solely by name; describe more complex techniques in the Methods section.*
☒	☐	A description of all covariates tested
☐	☒	A description of any assumptions or corrections, such as tests of normality and adjustment for multiple comparisons
☐	☒	A full description of the statistical parameters including central tendency (e.g. means) or other basic estimates (e.g. regression coefficient) AND variation (e.g. standard deviation) or associated estimates of uncertainty (e.g. confidence intervals)
☐	☒	For null hypothesis testing, the test statistic (e.g. *F*, *t*, *r*) with confidence intervals, effect sizes, degrees of freedom and *P* value noted<br>*Give P values as exact values whenever suitable.*
☒	☐	For Bayesian analysis, information on the choice of priors and Markov chain Monte Carlo settings
☒	☐	For hierarchical and complex designs, identification of the appropriate level for tests and full reporting of outcomes
☐	☒	Estimates of effect sizes (e.g. Cohen's *d*, Pearson's *r*), indicating how they were calculated

Our web collection on statistics for biologists contains articles on many of the points above.

Software and code

Policy information about availability of computer code

Data collection	NextSeq 500 platform (Illumina); NovoCyte Quanteon
Data analysis	Generated reads were quality-checked using FastQC (v0.11.8) and adapters were trimmed using Cutadapt (v1.16) with Python (v3.6.6), using the following parameters: -j 6 -a Illumina Read 1 adapter=AAGATCGGAAGAGCACACGTCTGAACTCCAGTCA -a Poly A=AAAAAAAAAAA --output=out1.fq.gz --error-rate=0.1 --times=1 --overlap=3 --minimum-length=20 --nextseq-trim=20 3_1<br>For both sequencing data types (dRNA-seq and conventional RNA-seq), READemption (v0.4.5) was used to map reads to the B. thetaiotaomicron VPI-5482 reference genome (NC_004663.1) and plasmid (NC_004703.1)<br><br>Transcriptome annotation: ANNOgesic pipeline (v0.7.33); sRNA database (https://github.com/Sung-Huan/ANNOgesic/tree/master/database); non-redundant (nr) protein database (ftp://ftp.ncbi.nih.gov/blast/db/FASTA/)<br><br>Prediction of invertible DNA regions (https://github.com/XiaofangJ/PhaseFinder): python PhaseFinder.py locate -f Bt_genome_plasmid.fa -t phasefinder.tab -g 15 85 -p<br><br>Differential gene expression analysis : R package edgeR (v3.38.2)<br><br>Gene set annotation and enrichment analyses: PULdb (http://www.cazy.org/PULDB/); RegPrecise v3.2, KEGG database (https://www.genome.jp/kegg/pathway.html, accessed on 01/Dec/22); GO terms from Uniprot (https://www.uniprot.org/, accessed on 25/Nov/21), eggNOG (v5.0.); fgsea R package (v1.27.0)<br><br>Theta-Base 2.0: Micromix (https://github.com/BarquistLab/Micromix); Gunicorn (https://readthedocs.org/projects/gunicorn-docs); Nginx

(https://www.nginx.com/)

Read Alignment visualization: JBrowse 2

In-silico interaction prediction : IntaRNA as part of the Vienna RNA package (2.4.14 and boost 1.7)

Flow cytometry: NovoExpress Software (v1.4.1)

Code for the Micromix data integration platform is available at https://github.com/BarquistLab/Micromix.

For manuscripts utilizing custom algorithms or software that are central to the research but not yet described in published literature, software must be made available to editors and reviewers. We strongly encourage code deposition in a community repository (e.g. GitHub). See the Nature Portfolio guidelines for submitting code & software for further information.

Data

Policy information about availability of data

All manuscripts must include a data availability statement. This statement should provide the following information, where applicable:

- Accession codes, unique identifiers, or web links for publicly available datasets
- A description of any restrictions on data availability
- For clinical datasets or third party data, please ensure that the statement adheres to our policy

Our analyzed sequencing data are accessible at http://micromix.helmholtz-hiri.de/bacteroides/. The raw sequencing data are available at NCBI Gene Expression Omnibus (http://www.ncbi.nlm.nih.gov/geo) under the accession number GSE234958.

Research involving human participants, their data, or biological material

Policy information about studies with human participants or human data. See also policy information about sex, gender (identity/presentation), and sexual orientation and race, ethnicity and racism.

Reporting on sex and gender	N/A
Reporting on race, ethnicity, or other socially relevant groupings	N/A
Population characteristics	N/A
Recruitment	N/A
Ethics oversight	N/A

Note that full information on the approval of the study protocol must also be provided in the manuscript.

Field-specific reporting

Please select the one below that is the best fit for your research. If you are not sure, read the appropriate sections before making your selection.

☒ Life sciences ☐ Behavioural & social sciences ☐ Ecological, evolutionary & environmental sciences

For a reference copy of the document with all sections, see nature.com/documents/nr-reporting-summary-flat.pdf

Life sciences study design

All studies must disclose on these points even when the disclosure is negative.

Sample size	Conventional RNA-seq of diverse growth conditions and dRNA-seq of pooled conditions were performed in biological duplicate. Similarly MAPS analysis was performed in biological duplicate. Testing for differential expression or enrichment was performed using a generalized linear model (GLM) likelihood ratio implemented in edgeR. Northern blots were performed in two biological replicates unless explicitly stated. EMSAs were performed in biological triplicate. RT-qPCR analysis was performed in a minimum of three biological replicates. For significant comparisons, a Mann-Whitney test was used. Growth curves were performed in a minimum of three biological replicates unless explicitly mentioned. Two plasmid assays were performed in biological triplicate. For significant comparisons, Tukey's multiple comparisons test was used. Minimal inhibitory concentration (MIC) strip assays were performed in a minimum of five biological replicates for doxycycline and three biological replicates for tetracycline. Sample sizes were chosen based on prior experience and previous studies.<br>No statistical method was used to predetermine sample size. No data were excluded from the analyses. The experiments were not randomized. The Investigators were not blinded to allocation during experiments and outcome assessment.
Data exclusions	No data were excluded from the analyses.
Replication	Conventional RNA-seq of diverse growth conditions and dRNA-seq of pooled conditions were performed in biological duplicate. Similarly MAPS analysis was performed in biological duplicate. Testing for differential expression or enrichment was performed using a generalized

linear model (GLM) likelihood ratio implemented in edgeR. Northern blots were performed in two biological replicates unless explicitly stated. EMSAs were performed in biological triplicate. RT-qPCR analysis was performed in a minimum of three biological replicates. For significant comparisons, a Mann-Whitney test was used. Growth curves were performed in a minimum of three biological replicates unless explicitly mentioned. Two plasmid assays were performed in biological triplicate. For significant comparisons, Tukey's multiple comparisons test was used. Minimal inhibitory concentration (MIC) strip assays were performed in a minimum of five biological replicates for doxycycline and three biological replicates for tetracycline. Sample sizes were chosen based on prior experience and previous studies.
No statistical method was used to predetermine sample size. No data were excluded from the analyses. The experiments were not randomized. The Investigators were not blinded to allocation during experiments and outcome assessment.

Randomization	The experiments were not randomized. Technical replicates and their respective controls were measured in parallel to minimize variations due to media batch, buffers, etc. while biological replicates were setup and measured independently from each other.
Blinding	The Investigators were not blinded to allocation during experiments and outcome assessment.

Reporting for specific materials, systems and methods

We require information from authors about some types of materials, experimental systems and methods used in many studies. Here, indicate whether each material, system or method listed is relevant to your study. If you are not sure if a list item applies to your research, read the appropriate section before selecting a response.

Materials & experimental systems

n/a	Involved in the study	
☒	☐	Antibodies
☒	☐	Eukaryotic cell lines
☒	☐	Palaeontology and archaeology
☒	☐	Animals and other organisms
☒	☐	Clinical data
☒	☐	Dual use research of concern
☒	☐	Plants

Methods

n/a	Involved in the study	
☒	☐	ChIP-seq
☐	☒	Flow cytometry
☒	☐	MRI-based neuroimaging

Plants

Seed stocks	N/A
Novel plant genotypes	N/A
Authentication	N/A

Flow Cytometry

Plots

Confirm that:

☒ The axis labels state the marker and fluorochrome used (e.g. CD4-FITC).

☒ The axis scales are clearly visible. Include numbers along axes only for bottom left plot of group (a 'group' is an analysis of identical markers).

☒ All plots are contour plots with outliers or pseudocolor plots.

☒ A numerical value for number of cells or percentage (with statistics) is provided.

Methodology

Sample preparation	Strains which were engineered to carry translational fusions of GFP were cultured in LB medium supplemented with chloramphenicol (20 µg/mL) and carbenicillin (100 µg/mL) until reaching an OD600 of 0.5. Subsequently, 100 µL of the cultures were harvested and subjected to three washes with 1x phosphate-buffered saline (PBS) prior to fixation with a 4% paraformaldehyde solution.
Instrument	NovoCyte Quanteon, Agilent

Software	NovoExpress Software (v1.4.1), GraphPad Prism version 9
Cell population abundance	All cells above a Threshold of 2000 were measured.
Gating strategy	The GFP fluorescence intensity of 100,000 cells above the Threshold of 2000 were measured.

☒ Tick this box to confirm that a figure exemplifying the gating strategy is provided in the Supplementary Information.
