# Supplementary material for: An expanded transcriptome atlas for Bacteroides thetaiotaomicron reveals a small RNA that modulates tetracycline sensitivity
Source: Nat Microbiol. 2024 Mar 25;9(4):1130–44. doi: 10.1038/s41564-024-01642-9 (PMC10994844; doi:10.1038/s41564-024-01642-9)

c)

BTnc207

Raw

Marker (nt)

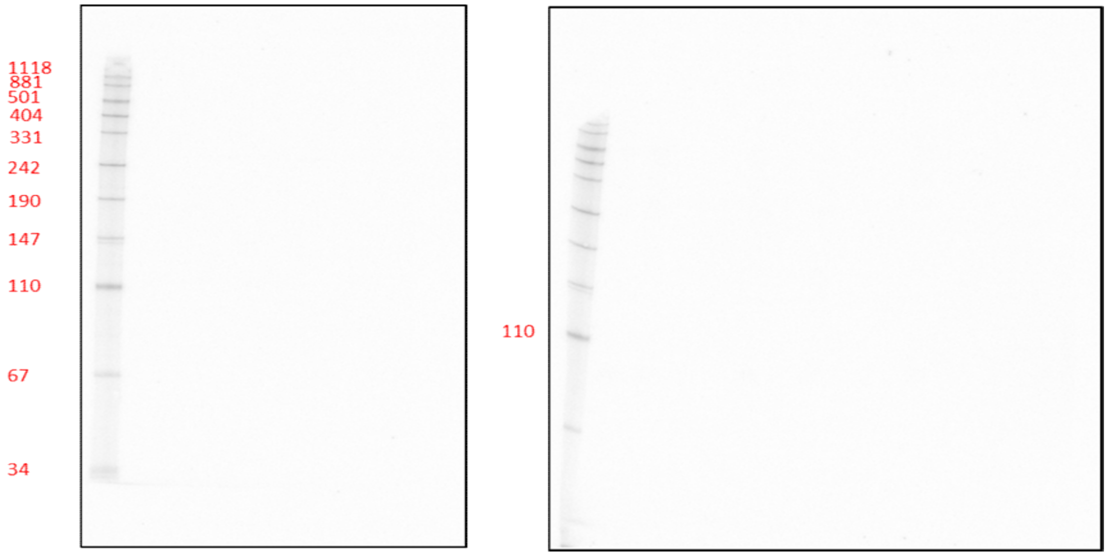

Brightness and contrast adjusted

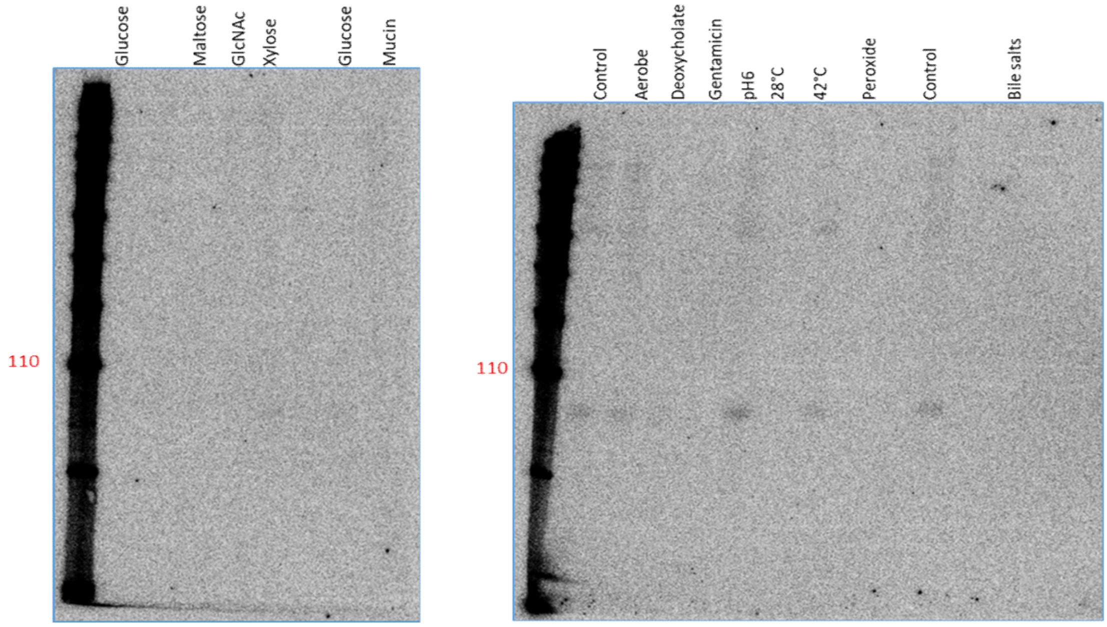

BTnc302

Raw

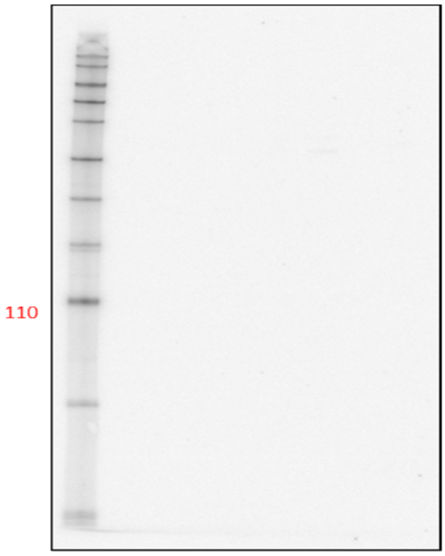

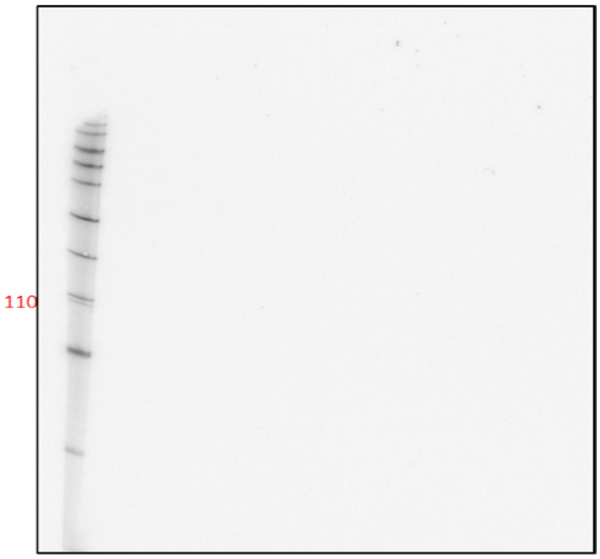

Brightness and contrast adjusted

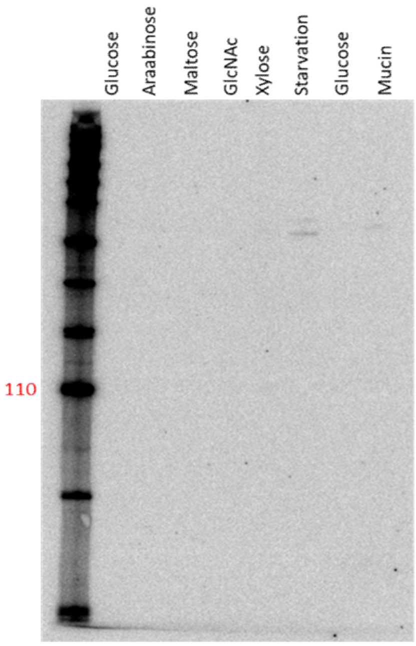

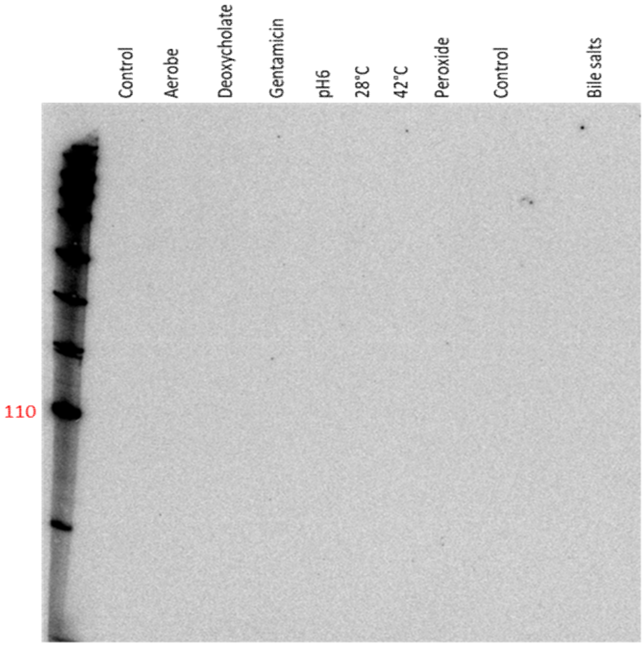

BTnc353

Raw

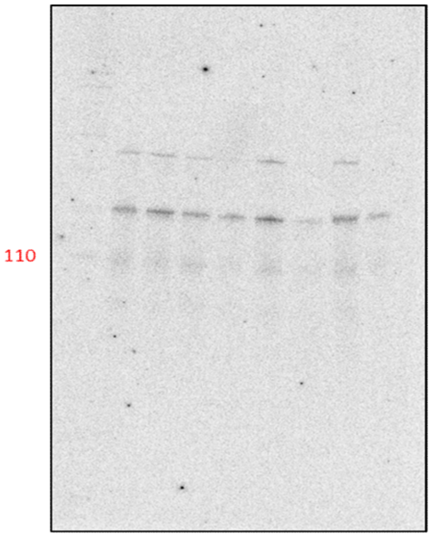

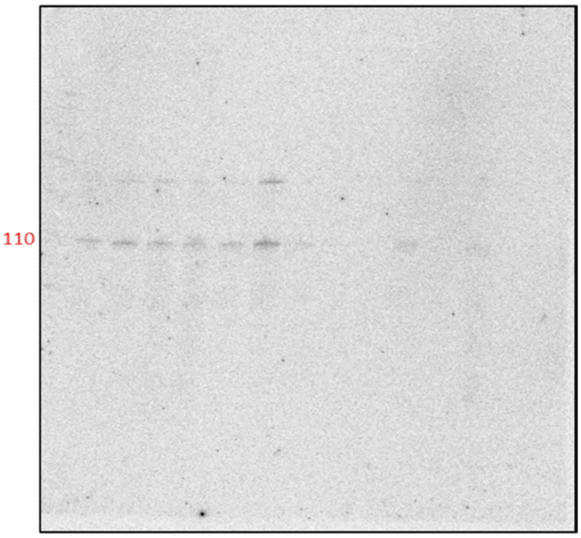

Brightness and contrast adjusted

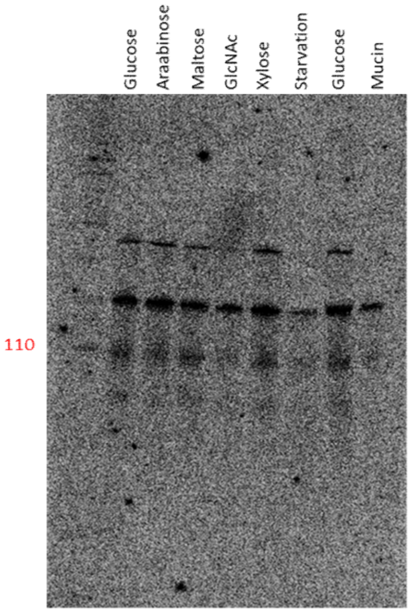

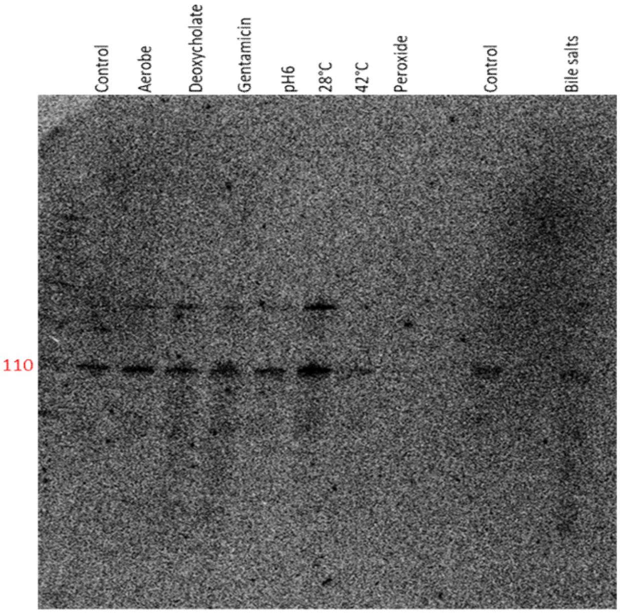

BTnc286

Raw

110

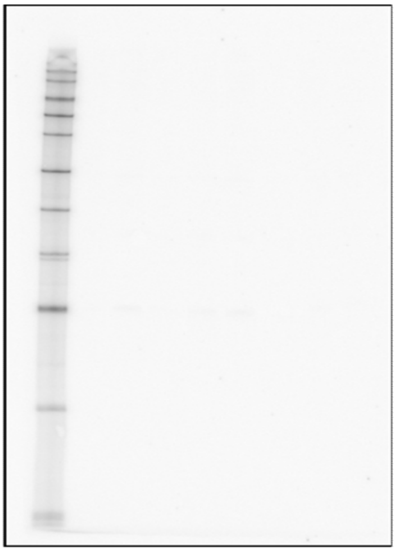

110

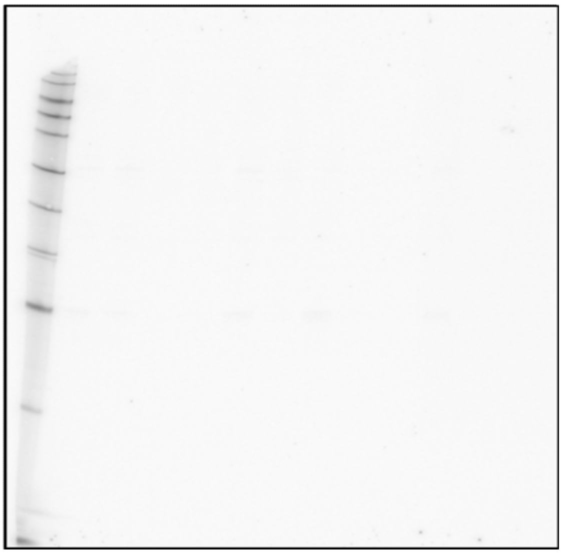

Brightness and contrast adjusted

110

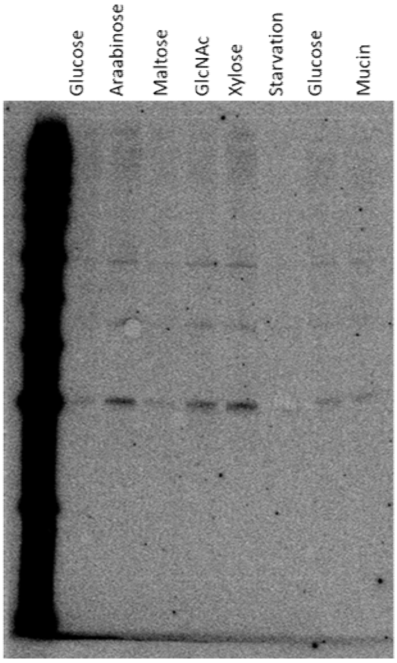

110

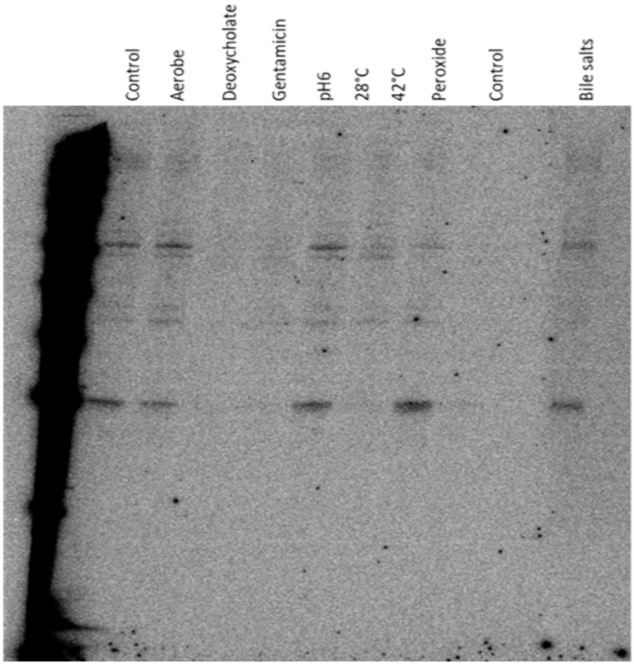

BTnc311

Raw

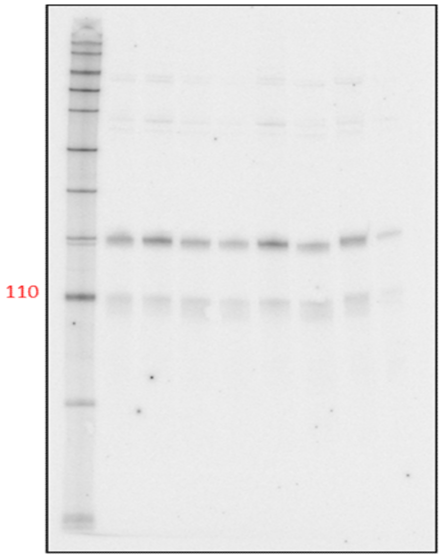

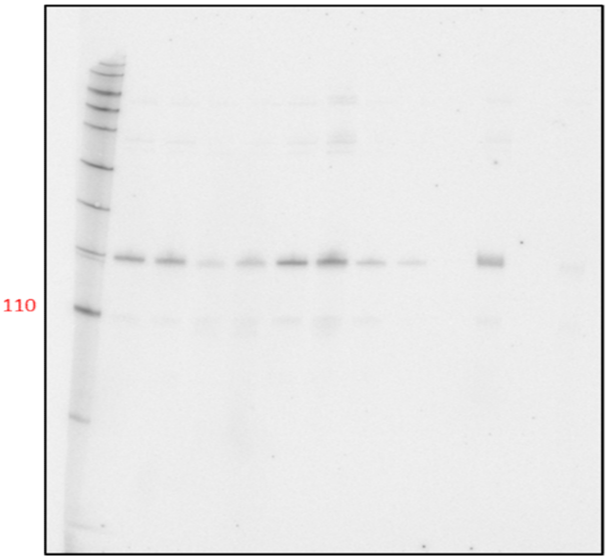

Brightness and contrast adjusted

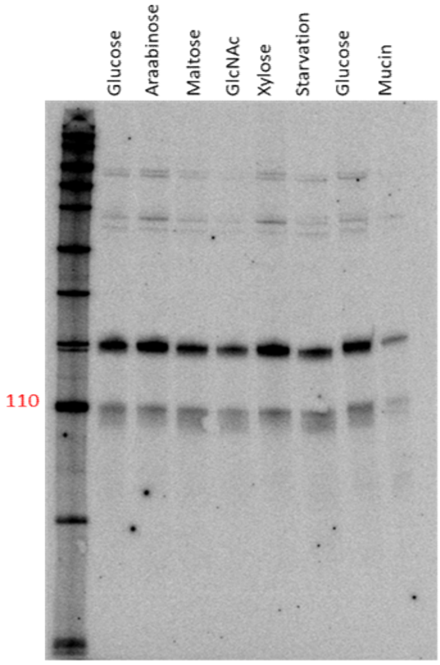

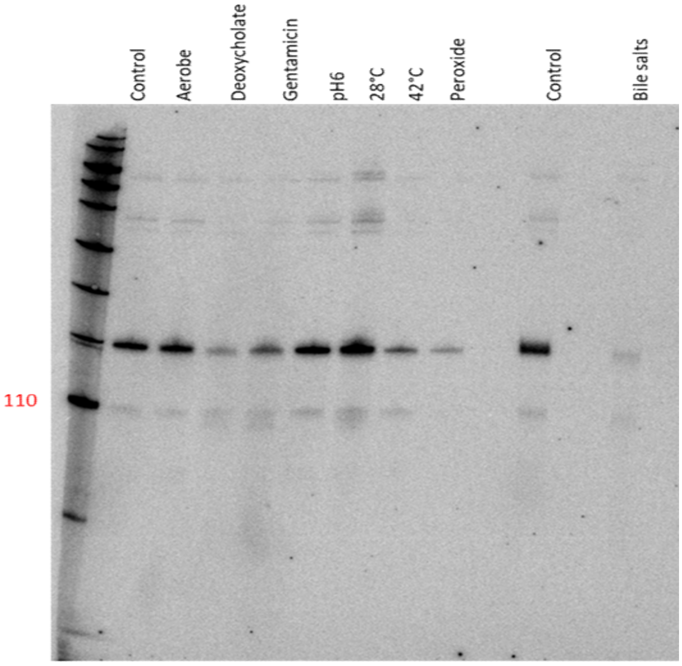

BTnc301

Raw

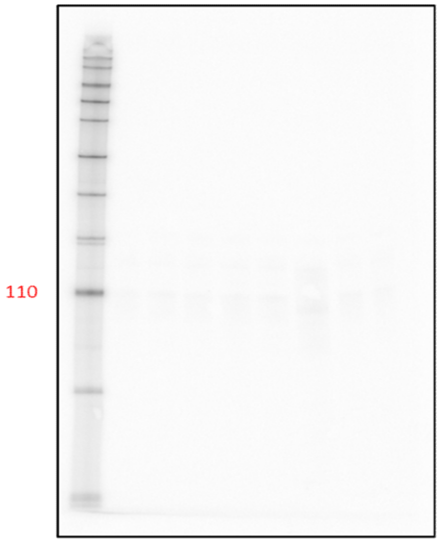

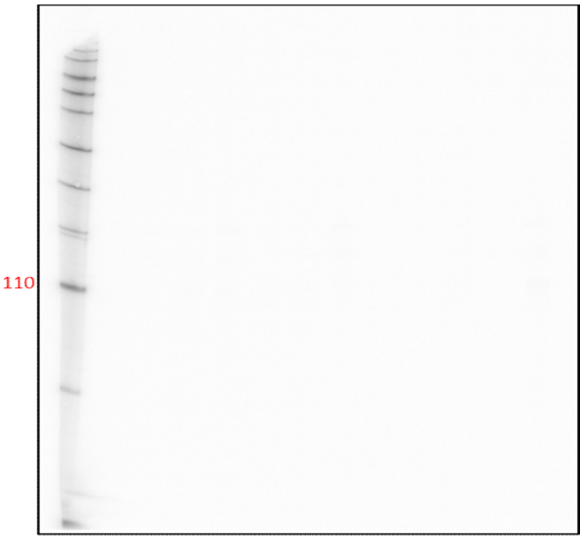

Brightness and contrast adjusted

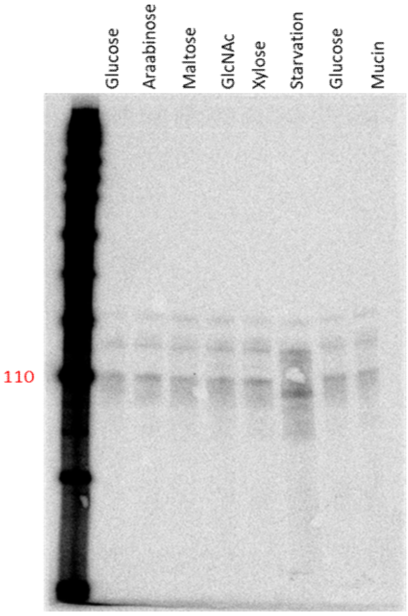

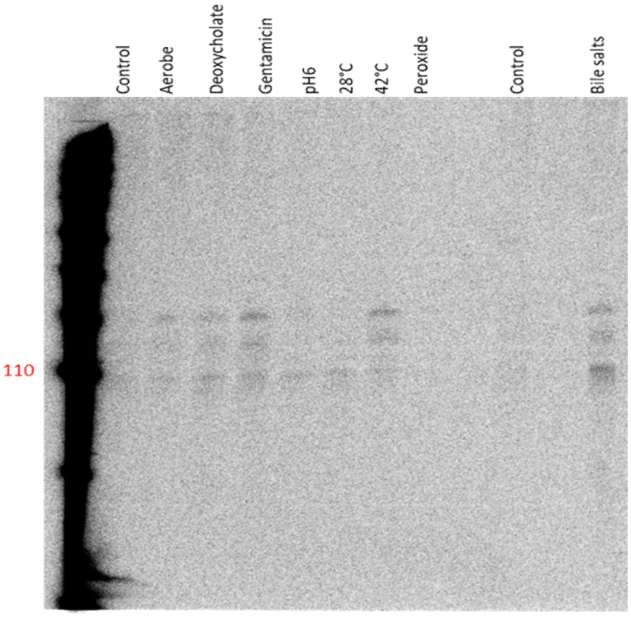

BTnc325

Raw

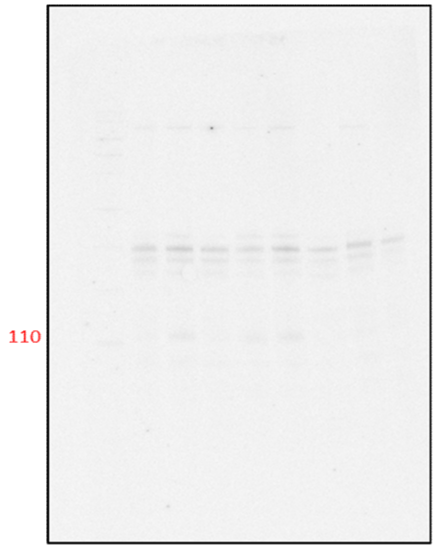

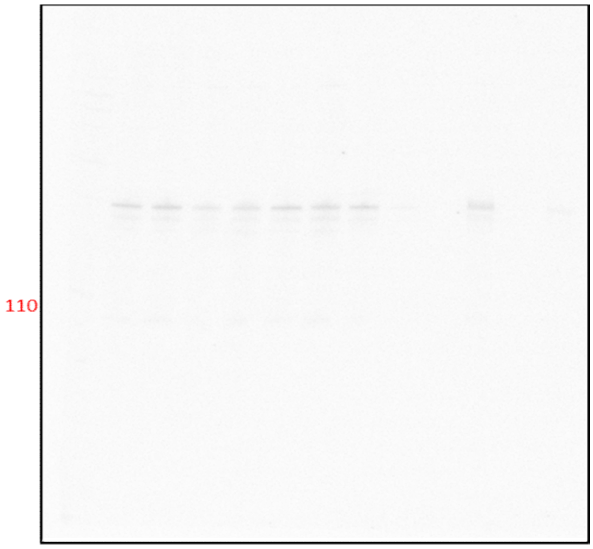

Brightness and contrast adjusted

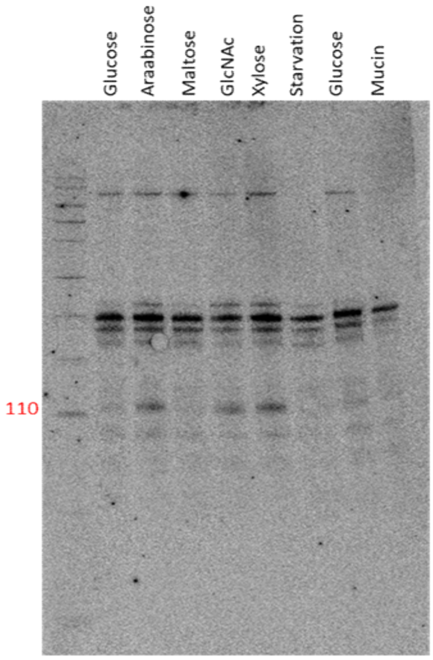

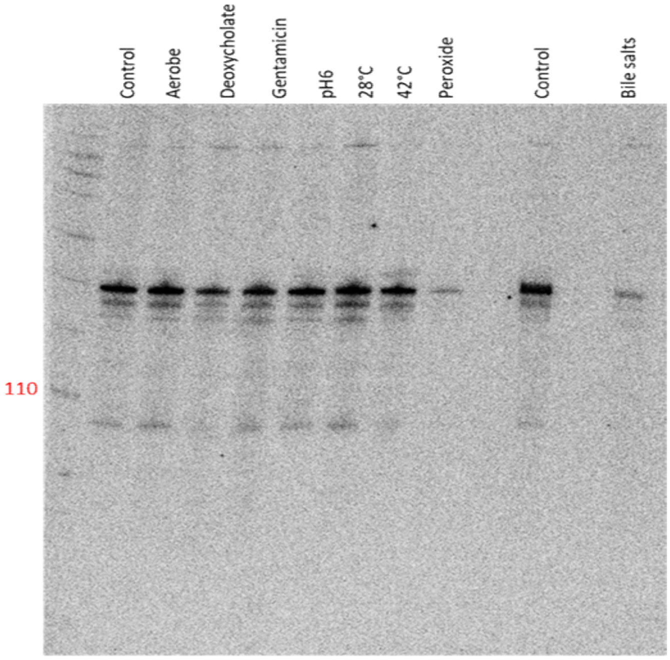

Supplement: Supplementary file 5 — Expression data for Fig. 3b. [file 41564_2024_1642_MOESM5_ESM.pdf]
