# Supplementary material for: An expanded transcriptome atlas for Bacteroides thetaiotaomicron reveals a small RNA that modulates tetracycline sensitivity
Source: Nat Microbiol. 2024 Mar 25;9(4):1130–44. doi: 10.1038/s41564-024-01642-9 (PMC10994844; doi:10.1038/s41564-024-01642-9)

b)

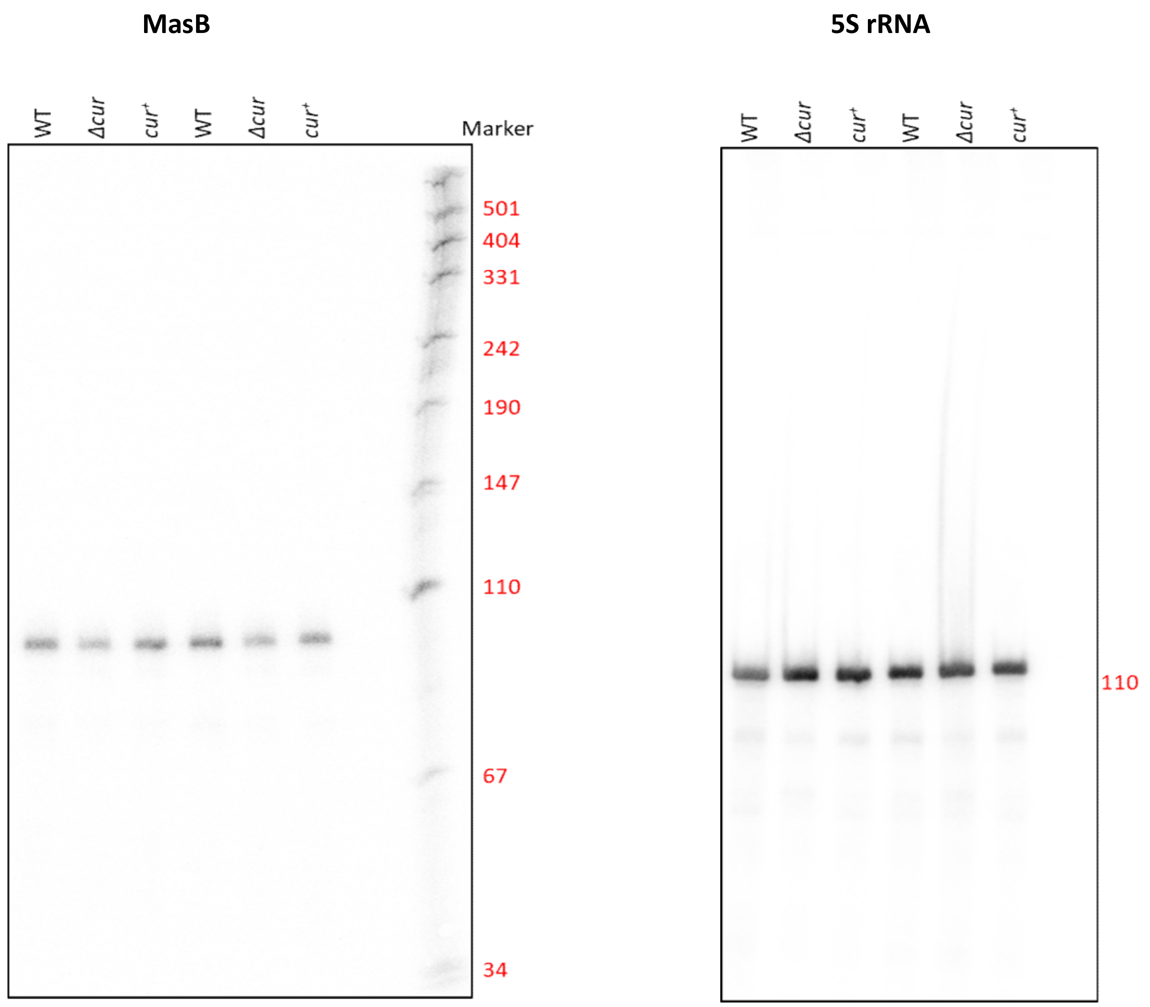
MasB
WT
Δcur
cur+
WT
Δcur
cur+
Marker
501
404
331
242
190
147
110
67
34
5S rRNA
WT
Δcur
cur+
WT
Δcur
cur+
110

e)

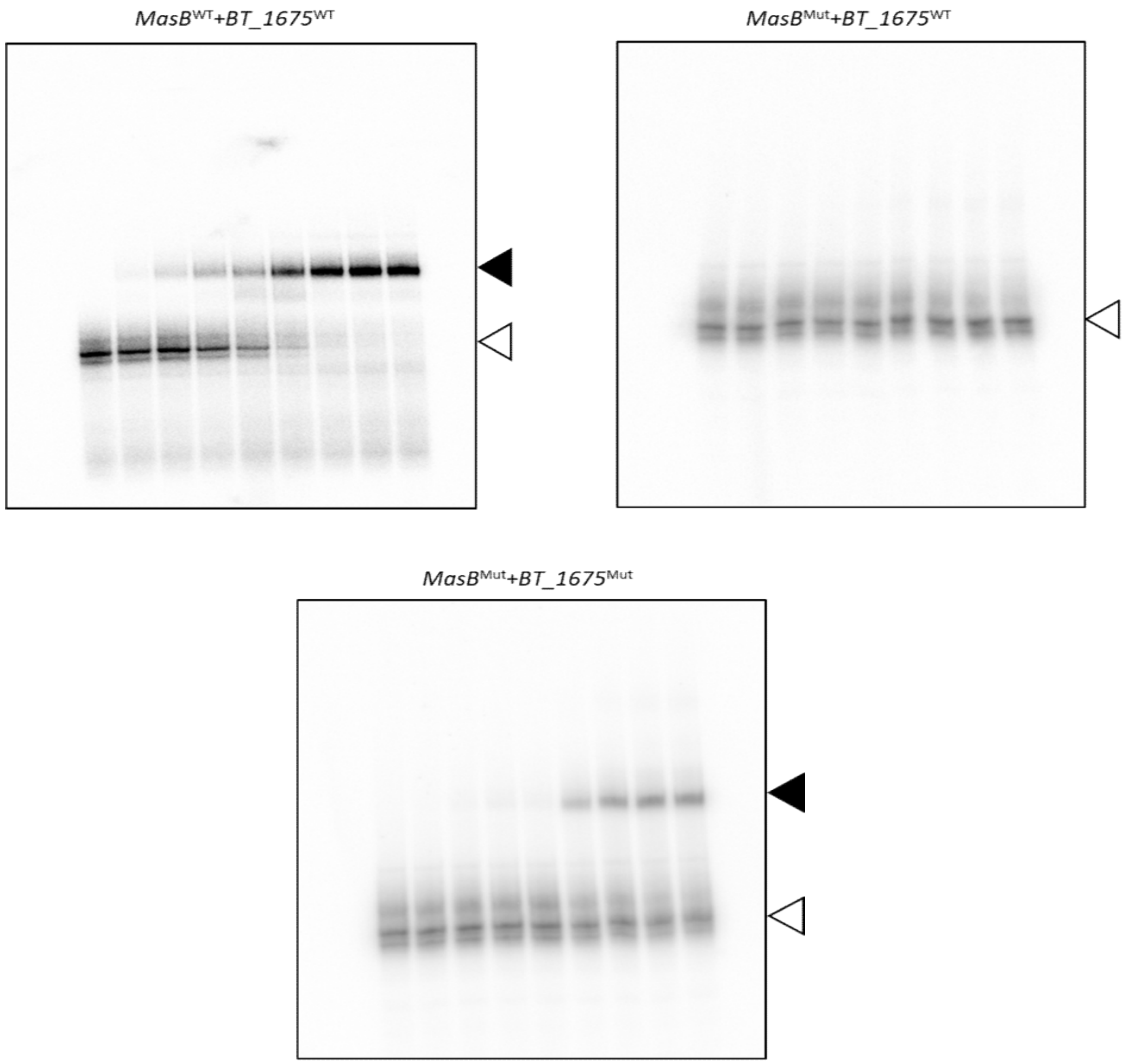

Supplement: Supplementary file 9 — Unmodified blots for Fig. 5b,e. [file 41564_2024_1642_MOESM9_ESM.pdf]
