# Supplementary figures and images for: An expanded transcriptome atlas for Bacteroides thetaiotaomicron reveals a small RNA that modulates tetracycline sensitivity
Source: Nat Microbiol. 2024 Mar 25;9(4):1130–44. doi: 10.1038/s41564-024-01642-9 (PMC10994844; doi:10.1038/s41564-024-01642-9)

b)

BTnc086

Raw

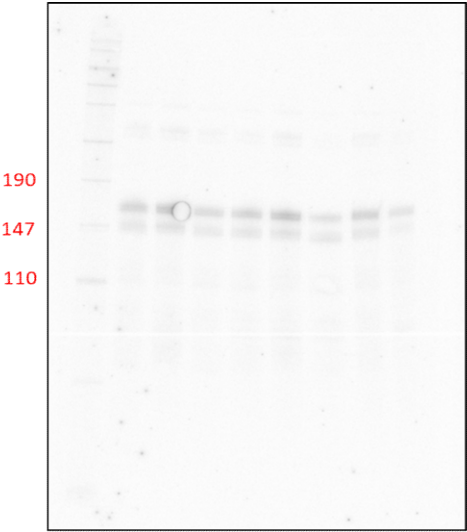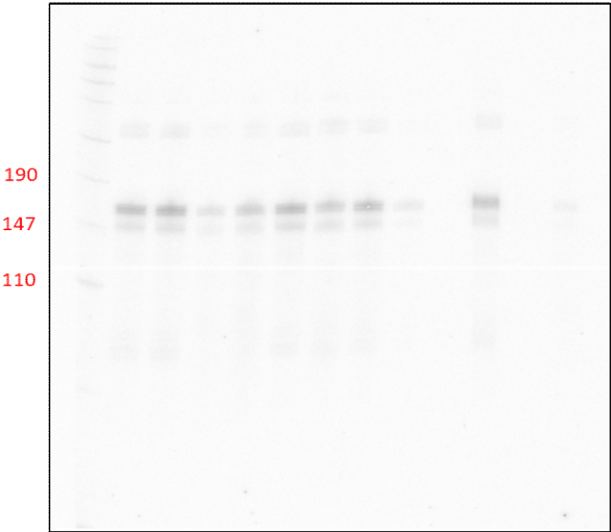

Brightness and contrast adjusted

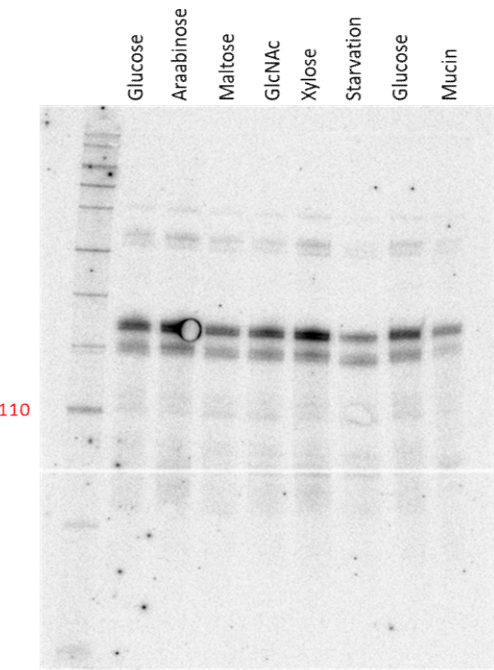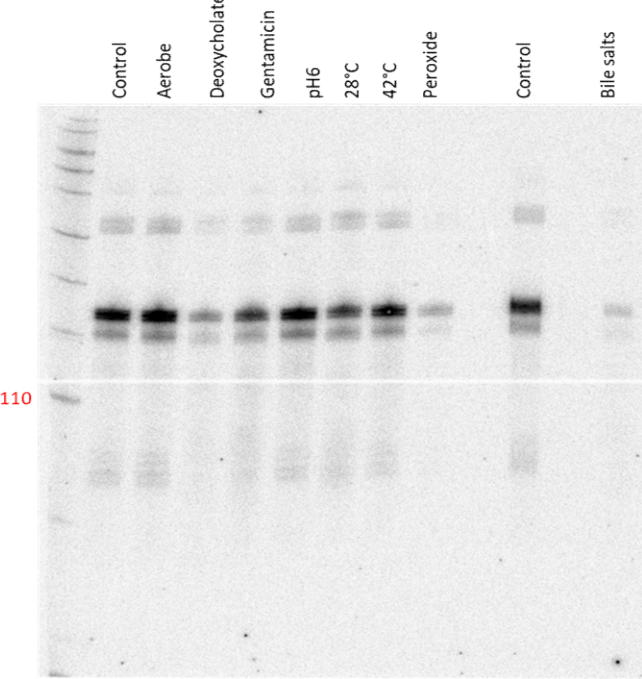

BTnc146

Raw

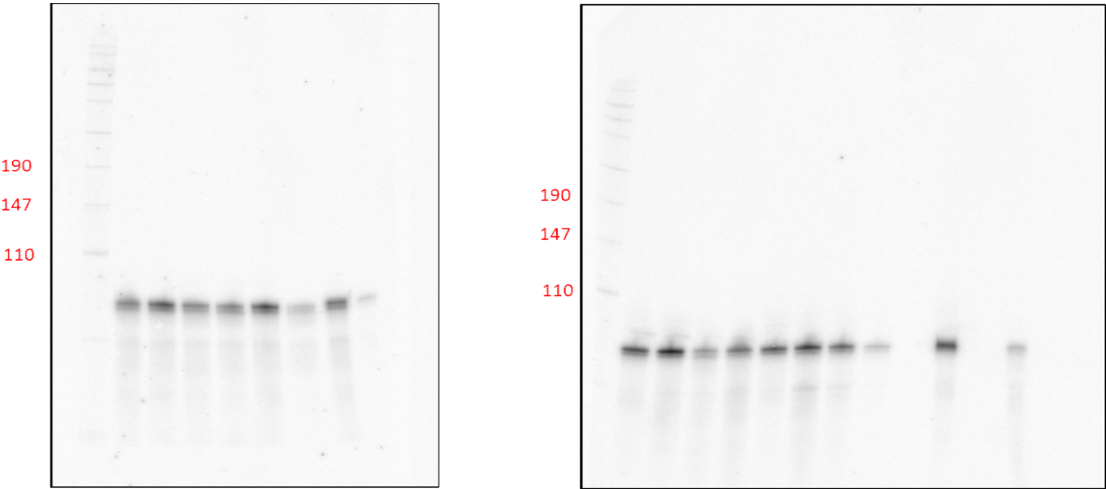

Brightness and contrast adjusted

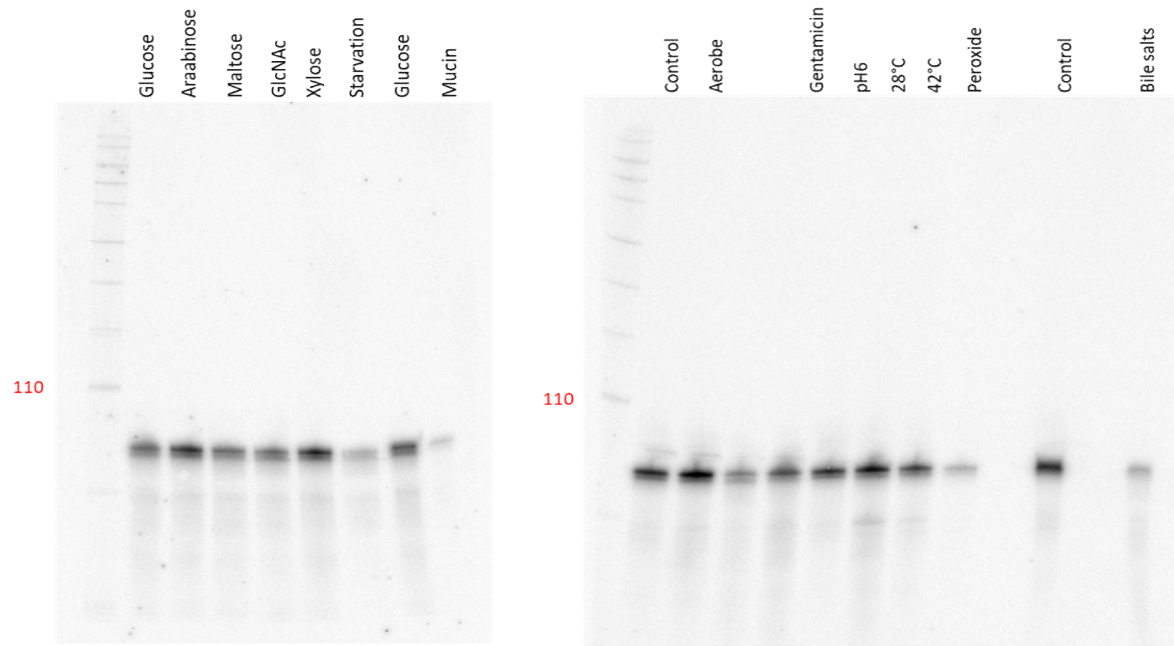

Supplement: Supplementary file 14 — Unmodified blots for Extended Data Fig. 7b. [file 41564_2024_1642_MOESM14_ESM.pdf]

d)

MasB

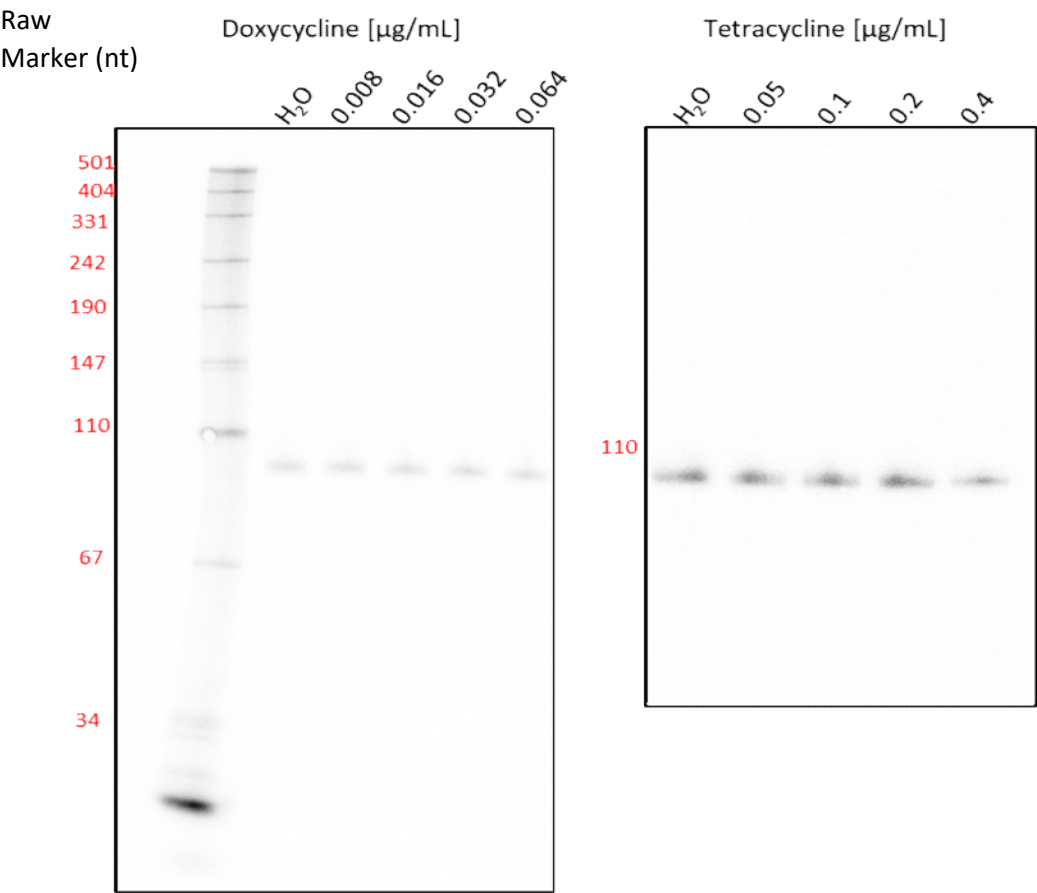

5S rRNA

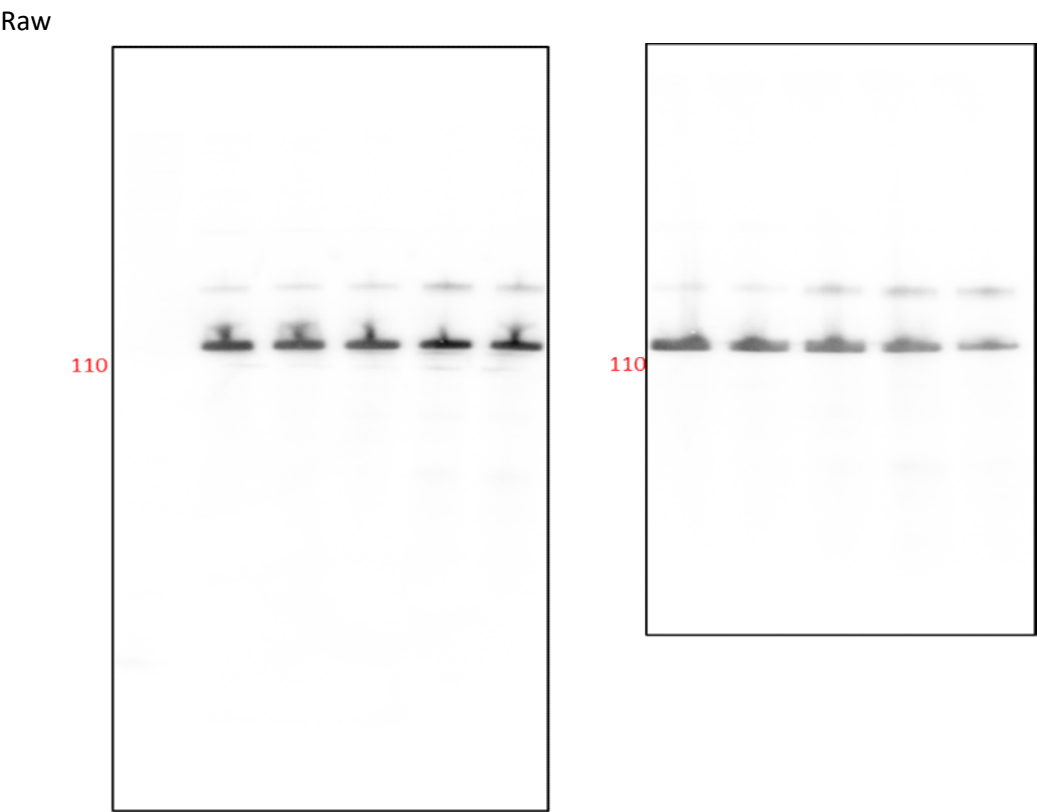

Supplement: Supplementary file 16 — Unmodified blots for Extended Data Fig. 8d. [file 41564_2024_1642_MOESM16_ESM.pdf]

d)  
Raw

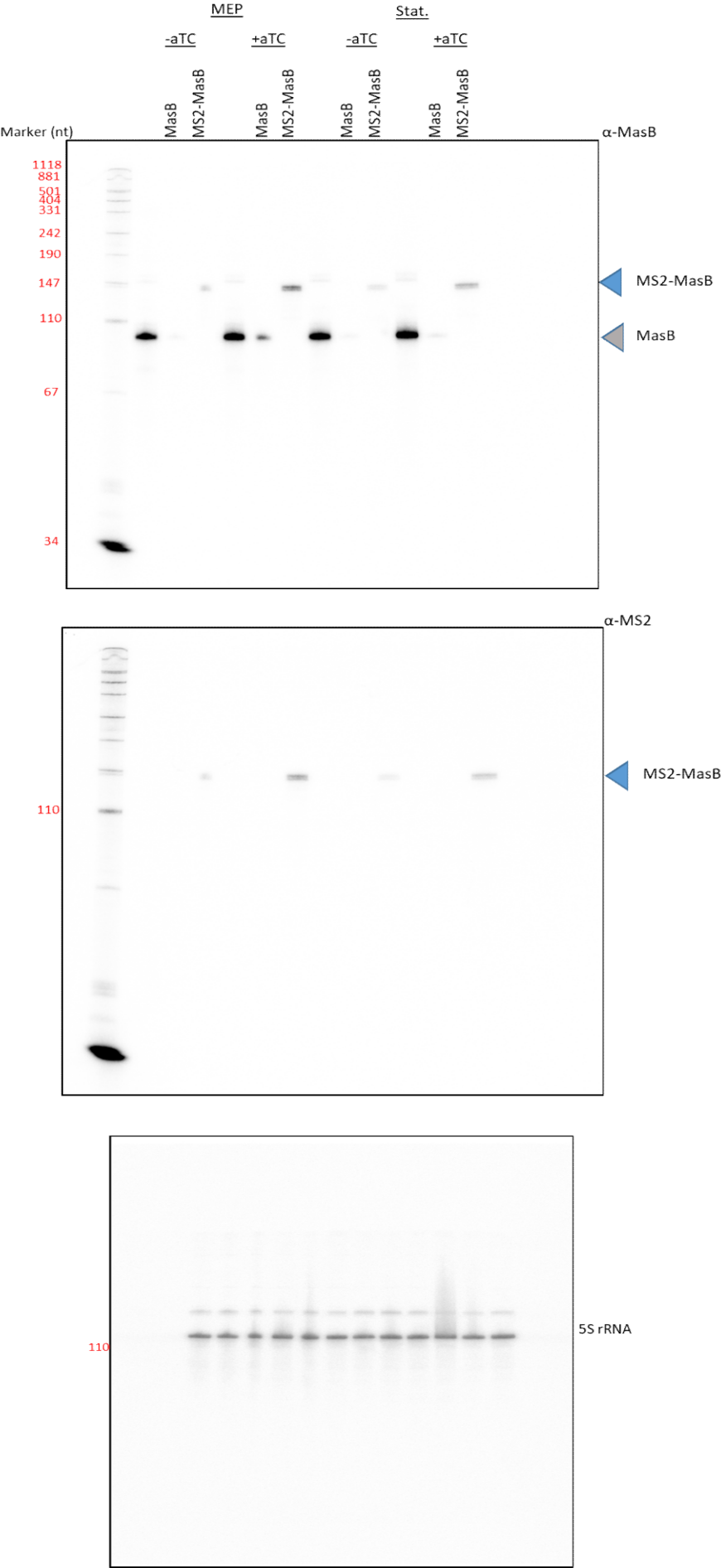

e)  
Raw

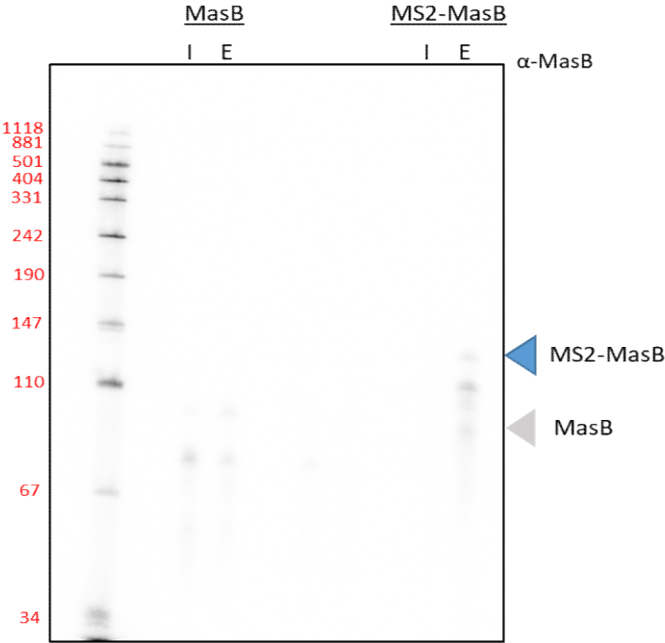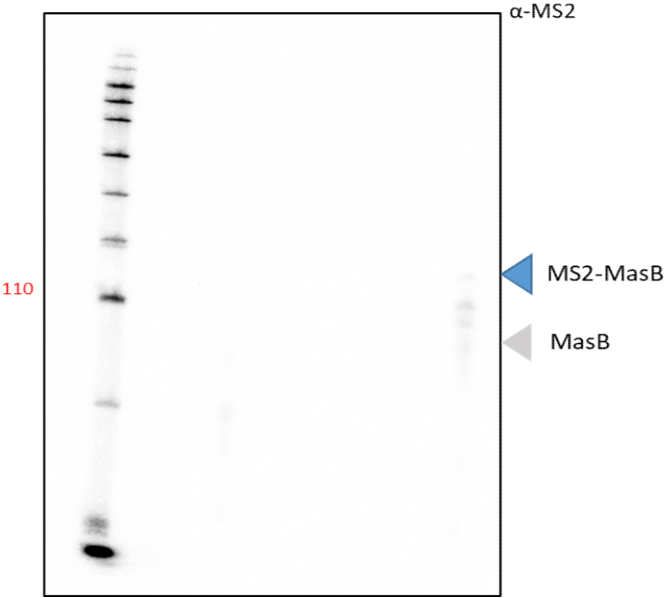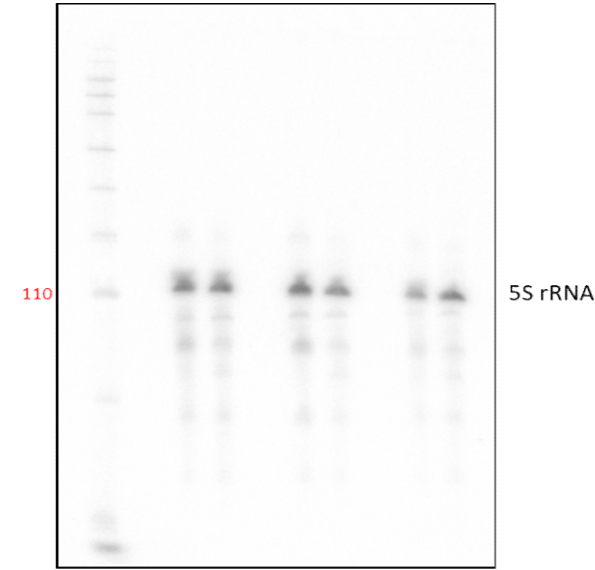

Supplement: Supplementary file 18 — Unmodified blots for Extended Data Fig. 9d,e. [file 41564_2024_1642_MOESM18_ESM.pdf]

c)

MasB

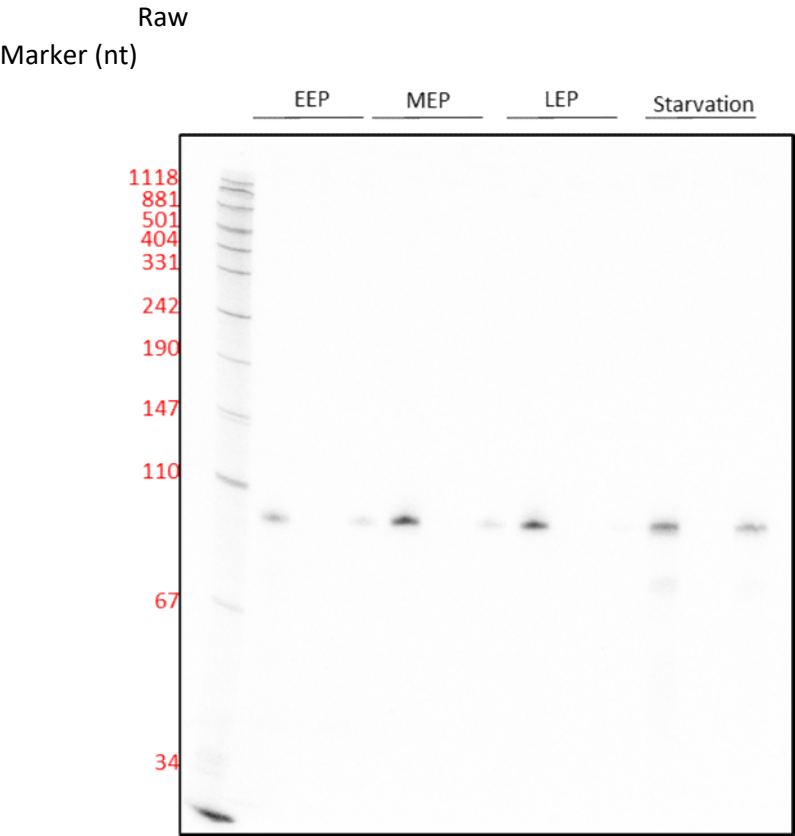

5S rRNA

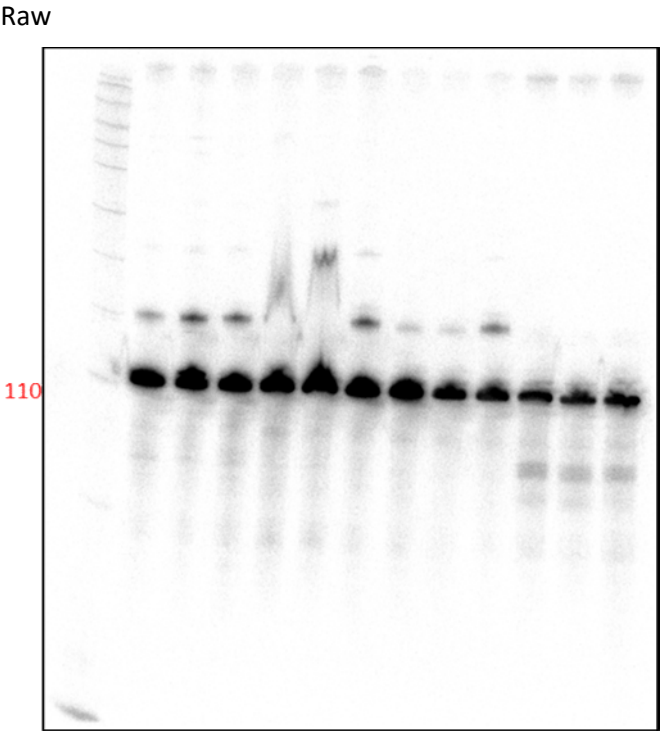

Supplement: Supplementary file 20 — Unmodified blots for Extended Data Fig. 10c. [file 41564_2024_1642_MOESM20_ESM.pdf]
